# Disparities in Cardiovascular Research Output and Disease Outcomes among High-, Middle- and Low-Income Countries – An Analysis of Global Cardiovascular Publications over the Last Decade (2008–2017)

**DOI:** 10.5334/gh.815

**Published:** 2021-01-18

**Authors:** Nada Qaisar Qureshi, Syed Hamza Mufarrih, Gerald S. Bloomfield, Wajeeha Tariq, Aysha Almas, Ali H. Mokdad, John Bartlett, Imran Nisar, Sameen Siddiqi, Zulfiqar Bhutta, Daniel Mark, Pamela S. Douglas, Zainab Samad

**Affiliations:** 1 Department of Medicine, The Aga Khan University, Karachi, PK; 2 Division of Cardiology, Department of Medicine, Duke University, Durham, NC, US; 3 Duke Clinical Research Institute, Duke University, Durham, NC, US; 4 Duke Global Health Institute, Duke University, Durham, NC, US; 5 Department of Health Metrics Sciences, University of Washington, Seattle, WA, US; 6 Department of Pediatrics and Child Health, The Aga Khan University, Karachi, PK; 7 Department of Community Health Sciences, The Aga Khan University, Karachi PK; 8 Centre of Excellence in Women and Child Health, Aga Khan University, Karachi, PK; 9 Centre for Global Child Health, The Hospital for Sick Children, Toronto, ON, CA; 10 University of Toronto, Toronto, ON, CA

**Keywords:** Bibliometrics, Cardiovascular disease burden, Low middle income countries, low income countries, global cardiovascular health

## Abstract

**Background:**

Cardiovascular disease (CVD) is the leading cause of death and disability worldwide. Health research is crucial to managing disease burden. Previous work has highlighted marked discrepancies in research output and disease burden between high-income countries (HICs) and low- and lower-middle-income countries (LI-LMICs) and there is little data to understand whether this gap has bridged in recent years. We conducted a global, country level bibliometric analysis of CVD publications with respect to trends in disease burden and county development indicators.

**Methods:**

A search filter with a precision and recall of 0.92 and 0.91 respectively was developed to extract cardiovascular publications from the Web of Science (WOS) for the years 2008–2017. Data for disease burden and country development indicators were extracted from the Global Burden of Disease and the World Bank database respectively.

**Results:**

Our search revealed 847,708 CVD publications for the period 2008–17, with a 43.4% increase over the decade. HICs contributed 81.1% of the global CVD research output and accounted for 8.1% and 8.5% of global CVD DALY losses deaths respectively. LI-LMICs contributed 2.8% of the total output and accounted for 59.5% and 57.1% global CVD DALY losses and death rates.

**Conclusions:**

A glaring disparity in research output and disease burden persists. While LI-LMICs contribute to the majority of DALYs and mortality from CVD globally, their contribution to research output remains the lowest. These data call on national health budgets and international funding support to allocate funds to strengthen research capacity and translational research to impact CVD burden in LI-LMICs.

## Introduction

Non-communicable diseases (NCDs), principally cardiovascular diseases (CVD), chronic respiratory diseases, cancer and diabetes, claim more than 41 million lives annually, accounting for 60–70% of global deaths [1,2,3,4,5]. According to the World Health Organization (WHO), in 2016, ischemic heart disease and stroke alone accounted for 15.2 million deaths globally, and have remained the leading causes of deaths in the last 15 years [6]. While NCDs have traditionally been associated with economic development, the epidemiologic transition has led to a double burden of communicable and non-communicable diseases in low and lower middle income countries. Estimates from 2012 suggest that 75% of the global mortality from NCDs occurred in low and lower middle income countries [4,7,8,9,10]. This double burden is a great impediment to low and lower middle income countries in meeting Sustainable Development Goal (SDG) targets [11,12].

Health research is critical to a coordinated response by countries to curb NCDs by applying principles of evidence-based medicine, health policy making, national and international funding allocation and programmatic development [8,13,14]. High income countries have fervently addressed the rising prevalence of CVD burden by using health research to discover disease pathogenesis, develop treatment, enhance health system infrastructures, track and improve outcomes, and target risk factors at person and population levels [15,16,17]. Such a trend has been less evident in the low- and lower-middle-income group [14,18]. Previous region-specific work has highlighted marked discrepancies in resource allocation to research and disease burden between the developed and developing world and there is little data to understand whether this gap has bridged in recent years [13,19,20,21,22,23,24,25,26,27]. Although not the absolute indicator, analyses of published research output is a reasonable way to measure health research and its impact.

We aimed to conduct a global, country-level bibliometric analysis of cardiovascular disease publications trends and understand the relationship of these trends with disease burden and country development indicators for the time period 2008–2017.

## Methods

We performed a bibliometric analysis of global cardiovascular publications, burden of disease and country development indicators over 10 years (2008–2017).

### Data Acquisition

#### Cardiovascular Publications

##### Filter development

A search filter with a precision and recall of 0.92 and 0.91 respectively was developed to extract cardiovascular publications from the Web of Science (WOS) for the years 2008 through 2017. The initial version of filter, which combined MeSH terms for diseases defined as cardiovascular disease by the WHO, had a precision and recall of 0.61 and 0.52 respectively.

Precision is the proportion of publications from the filter output that qualify as truly relevant to cardiovascular disease and recall is the proportion of known cardiovascular publications retrieved by the filter [13,28]. To improve recall, relevant words/phrases need to be added to the search term. To improve precision, irrelevant words/phrases need to be removed from the search filter. A reference set of cardiovascular publications was curated through manual review of articles published in top ranking cardiovascular journals by four independent reviewers. A list of relevant terms to add was generated through the frequency analysis of the titles and abstracts of articles in the reference set. A list of irrelevant terms to remove to improve precision was generated through the frequency analysis of the titles and abstracts of the irrelevant articles found in the random sample taken from the search results from the preceding filter. Word and phrase frequency analysis was conducted using TAPoR- Text analysis Portal for Research and TerMine from the National Center for Text Mining (NaCTeM) respectively.

##### Filter Application

The final filter was applied to the Science Citation Index Expanded [SCI-EXPANDED] and Social Sciences Citation Index [SSCI] in WOS for the years 2008 through 2017. WOS provides de-duplicated results for publications appearing in both editions. All types of documents except editorial material, letters, news items, proceeding papers and retractions were included in the analysis. No language filters were applied to the search. Results for each year were analyzed using WOS to produce country wise distribution of cardiovascular publications (Appendix A).

##### Country Participation and international collaboration

Country participation provided by the WOS is a reflection of integer counts, where each unique country contribution per publication is counted in full. Fractional counts assign country participation as a fraction by dividing the number of authors from one country by the total number of authors on the paper. Based on prior work where integer counts were used to make country level comparisons in research output, we used integer counts for our analyses [13,25,26,27]. A sensitivity analysis for WOS generated output was conducted through manual extraction of fractional and integer counts from using random samples of 383 articles (95% Confidence Interval) each from the years 2010 and 2015. The fractional contribution estimate (ratio of fractional to integer counts) has previously been used as a measure of international collaboration [13]. Results were used to calculate the fractional contribution estimates to reflect the degree of international collaboration in 2010 and 2015 (Appendix B).

#### Cardiovascular disease burden and country development indicators

Indicators for the years 2008 through 2017 were extracted from publicly available data. The Global Burden of Disease results tool was used to extract country level data for age-standardized prevalence (Appendix C), disability adjusted life years (DALYs) (Appendix D) and death rates (Appendix E) due to cardiovascular disease (ICD-10 and ICD-9 codes in Appendix I). The World Bank database was used to extract population numbers (Appendix F) and Human development index (HDI) (a combination of life expectancy, literacy rates and Gross National Income per capita) (Appendix G) for each country.

### Data cleaning

Country level data for publications, burden of disease and country development indicators were organized into the four World Bank income groups (2019) based on per capita income: low income (less than $996) lower-middle income ($996–$3895), upper-middle income ($3896–$12,055), and high income (greater than $12,055). Countries with data missing for either burden of disease or development indicators were excluded from the analysis.

### Comparisons and Analyses

Absolute publication counts, trends and percentage increase (*[publications in 2017- Publications in 2008]/publications in 2008*) in publication counts from 2008 to 2017 for cardiovascular research were extracted as provided by the Web of Science. Total counts, trends and percentage increase in the number of publications over the 10 years were calculated for each income group using integer counts. Within each income group, the top 10 countries were ranked by their percent contribution to the total cardiovascular research output of the whole group and percentage increases in their number of publications from 2008–2017 were then calculated. Publication rates per 100,000 population for each country were calculated to be depicted on the world map.

DALY and death numbers due to cardiovascular disease in each income group were reported as a percentage of global cardiovascular DALY and death numbers. Country level comparisons of prevalence, DALY, death rates and Human Development Index (HDI) to publication counts were done for the mid-study year (2013). Trends in publication numbers based on integer counts were plotted with the trends in combined prevalence, DALY and death rates due to cardiovascular disease over the 10 years for each income group. SPSS was used to calculate p values for significance in changes in trends. Bonferroni adjustment was applied and threshold for significance was p < 0.05. An exponential smoothing model was used to forecast trends till the year 2025 [29,30,31,32,33,34,35,36]. To assess the accuracy of the forecast, data from the first five years (2008–2012) was used to forecast numbers for the next five years (2013–2017) and compared this forecast to the actual data. Assessment of forecast accuracy revealed forecasted data for the years 2013–2017 to be within the 99% confidence intervals of the actual data.

The differences in measures of central tendencies for prevalence, DALYs and death rates between 2008 and 2017 were analyzed for significance for the four income groups. The Shapiro-Wilk test of normalcy was applied to check if data were normally distributed to select the appropriate measure of central tendency. The t-test was applied to means and the Mann-Whitney U test was used to analyze the difference in medians. Analyses were performed using Spss v. 22.

#### Role of funding source

The content of this paper is solely the responsibility of the authors and the funders have played no role in the study design, analysis, writing or decision to publish.

#### Data availability

All relevant data have been attached as supplementary files.

## Results

A total of 181 countries from the high (n = 52), upper middle (n = 52), lower middle (n = 44) and low (n = 33) income groups were included in the analysis.

### Publications and Publication Trends

**Figure 1:**
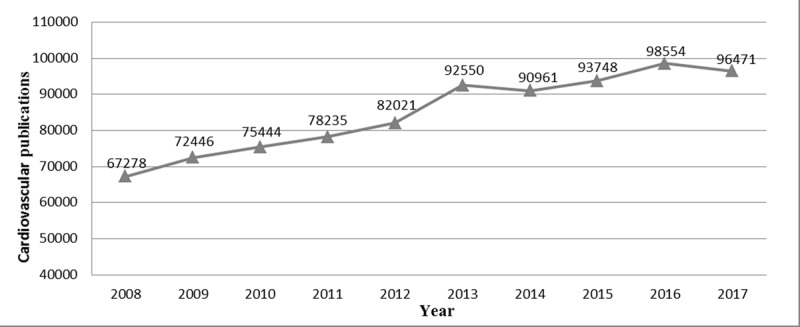
Absolute counts of cardiovascular publications (2008–2017).

**Figure 2:**
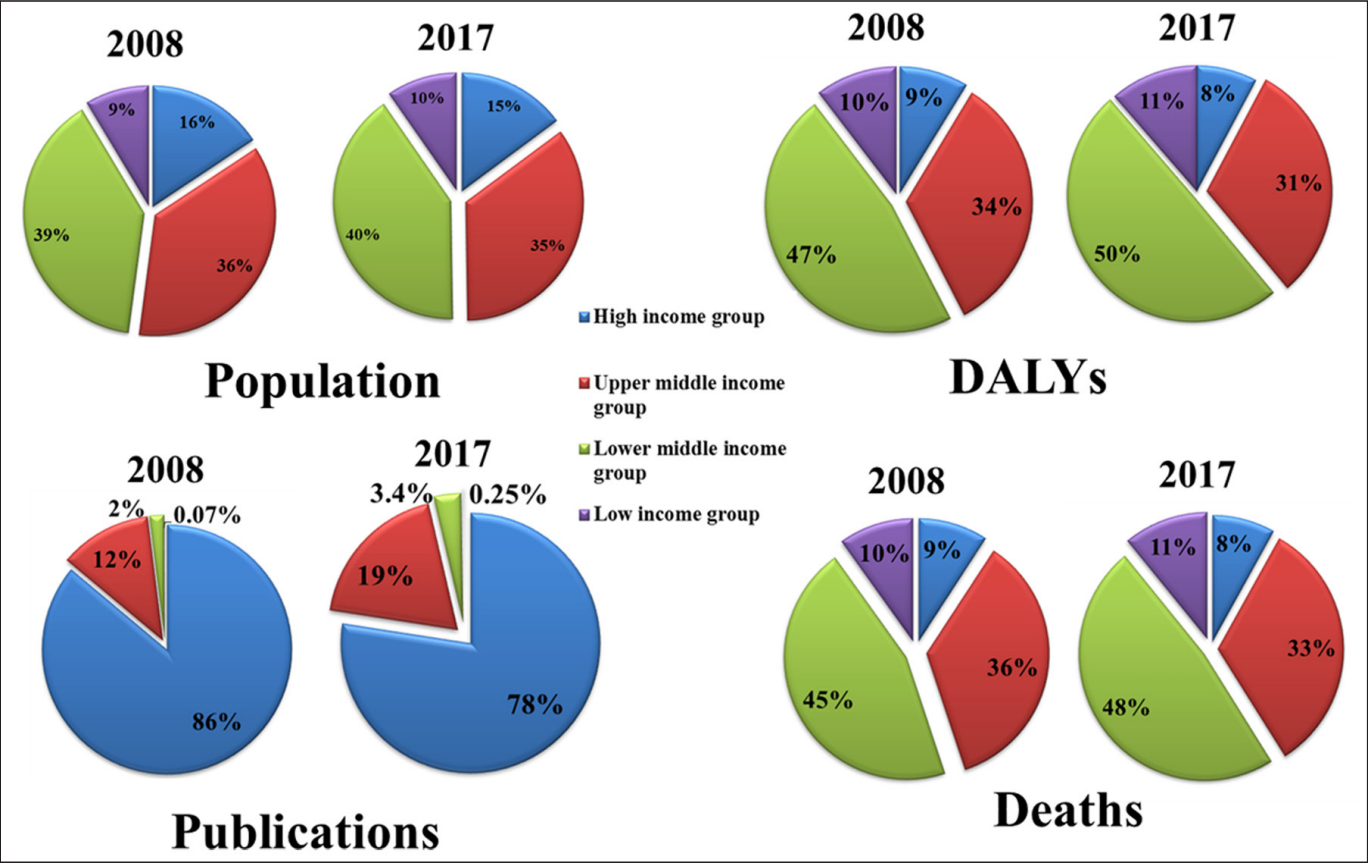
Population, cardiovascular DALY, deaths and publications in World Bank income groups as percentages of global figures.

The total global cardiovascular publication output for the time period 2008–2017 totaled 847,708 publications. Cardiovascular research output increased steadily over the decade, with a 43.4% increase in absolute counts of cardiovascular publications in 2017 compared to 2008 (Figure 1). Using integer counts, the contribution to global cardiovascular research output by high income was 81.1% and upper-middle, lower-middle and low-income countries contributed 16.1%, 2.6% and 0.2% respectively (Table 1, Figure 2). The fractional contribution estimate for total cardiovascular publications decreased from 0.89 in 2010 to 0.84 in 2015, reflecting an increase in the level of international collaboration (Appendix B).

**Table 1:** Cardiovascular publication integer counts for CVD in World Bank income groups as percentages of global figures.

Year	High income group	Upper middle income group	Lower middle income group	Low income group

**No. of countries**	52	52	44	33
**2008**	63,111 (86%)	8,751 (11.9%)	1,446 (1.9%)	55 (0.07%)
**2009**	68,821 (85.5%)	9,971 (12.4%)	1,649 (2%)	65 (0.08%)
**2010**	69,402 (83.1%)	12,204 (14.6%)	1,832 (2.2%)	99 (0.12%)
**2011**	73,240 (83.5%)	12,335 (14.1%)	1,990 (2.3%)	96 (0.11%)
**2012**	77,132 (8.6%)	13,722 (14.7%)	2,366 (2.5%)	132 (0.14%)
**2013**	86,401 (80.8%)	17,777 (16.6%)	2,567 (2.4%)	136 (0.13%)
**2014**	81,871 (79.8%)	17,640 (17.2%)	2,884 (2.8%)	164 (0.16%)
**2015**	85,639 (78.6%)	20,018 (18.4%)	3,052 (2.8%)	208 (0.19%)
**2016**	89,828 (78.2%)	20,801 (18.1%)	3,927 (3.4%)	253 (0.22%)
**2017**	89,370 (77.4%)	21,856 (18.9%)	3,960 (3.4%)	288 (0.25%)
**Total**	784,815 **(81.1%)**	155,075 **(16.1%)**	25,673 **(2.65%)**	1,496 **(0.15%)**

**Figure 3:**
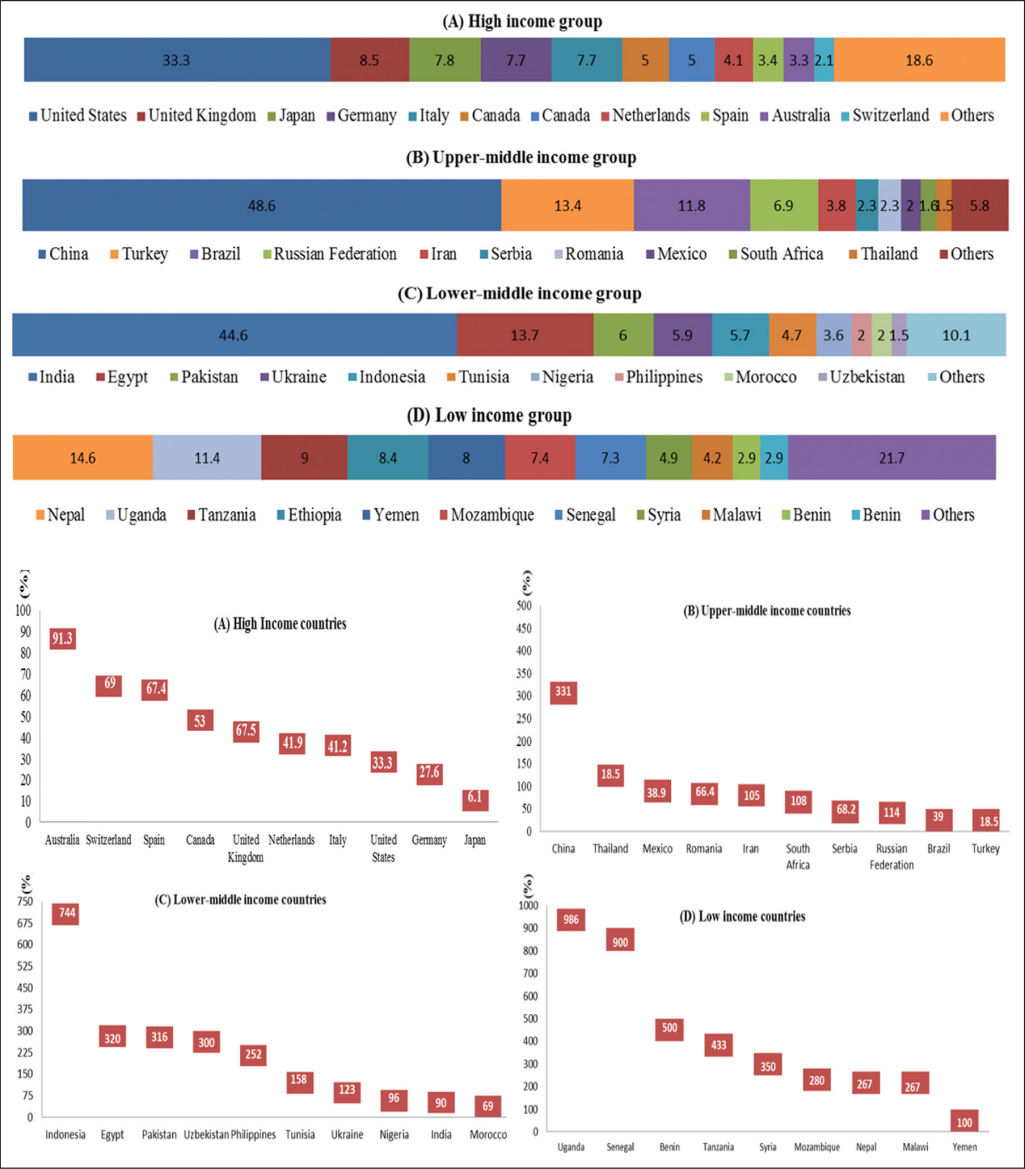
**(i)** Ranking of top 10 countries based on contribution to the 10-year cardiovascular research output of each income group. The width of the bar represents magnitude of contribution. Numbers for each country are its CV publications integer counts as a percentage of total CV publication integer counts of its income group. **(ii)** Percentage increase in integer counts of cardiovascular publications in top 10 countries based on contribution to total research output of each income group from 2008 to 2017.

The United States has the highest number of publications worldwide and contributed 27% of the global cardiovascular research output. Among high income countries, United States was the major contributor of cardiovascular research output across the decade (33.2%) but Australia had the highest percentage increase (91.3%) in publication numbers in 2017 compared with 2008 (Figure 3(i)A and 3(ii)A). Among the upper-middle-income countries, China contributed to the majority of research output (48.6%) and also had a three-fold increase (331.0%) in its publication numbers over the course of 10 years (Figure 3(i)B and 3(ii)B). Among low-middle-income countries, India contributed the major share of research output (48.6%), while Indonesia had a seven-fold increase (744.1%) in publication counts (Figure 3(i)C and 3(ii)C). Nepal contributed the most publications (14.6%) to the low income group in the 10 years with Ethiopia showing the highest percentage increase (2050.3%) in publication counts in 2017 compared with 2008 (Figure 3(i)D and 3(ii)D).

**Figure 4:**
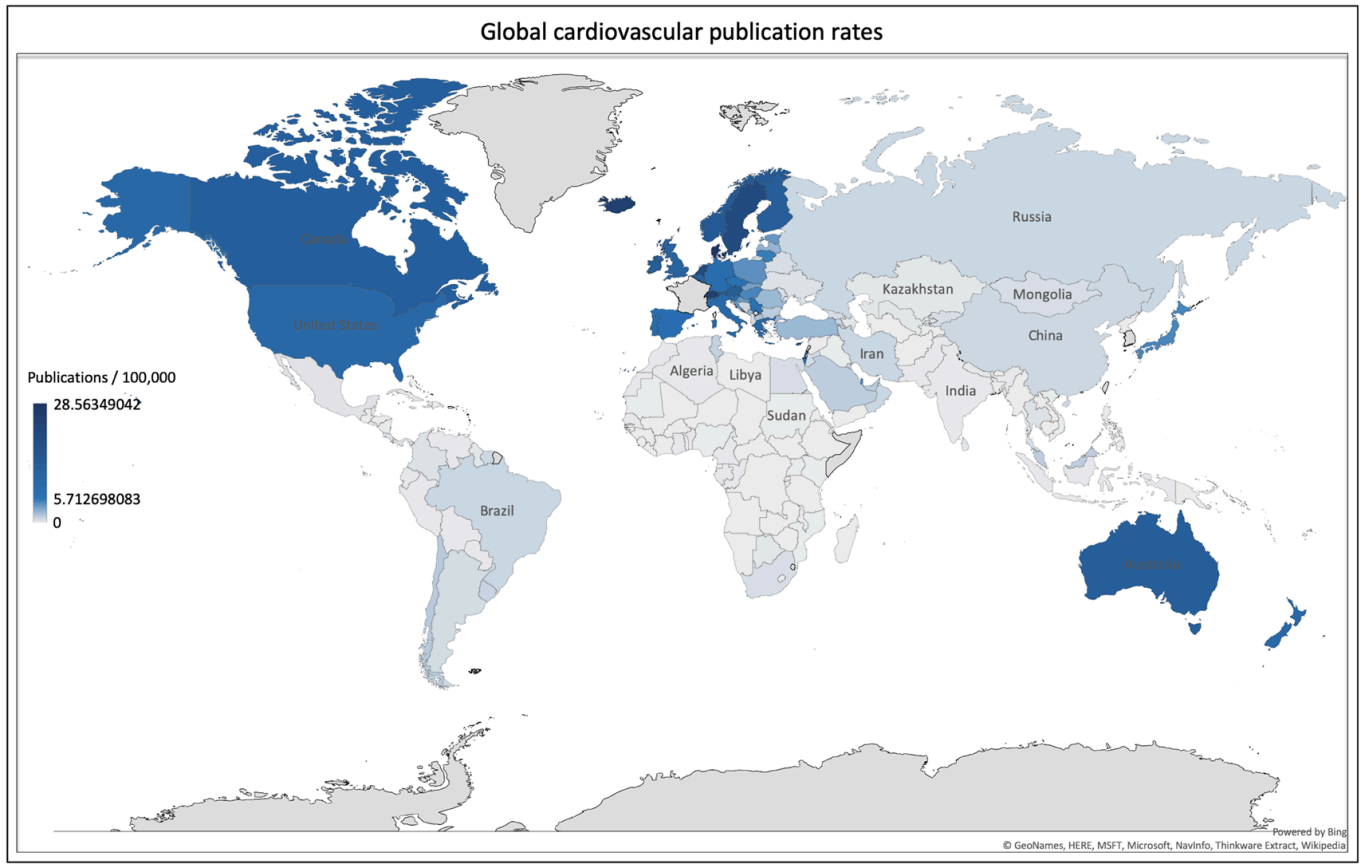
Hover over heat-map showing publication rates per 100,000 population based on integer counts (attached).

Although the United States accounted for the highest total number of cardiovascular publications for the 10 years, publication rates per 100,000 population were the highest for Denmark at 28.6 publications/100,000. Other countries with high publication rates included Iceland (24.6), Switzerland (24.5), Sweden (20.2), Norway (14.9), Austria (14.1), Ireland (14.0), Canada (12.6), United Kingdom (11.8) and United States (8.9) (Figure 4).

### Human Development Index (HDI) and Cardiovascular Research Output

**Figure 5:**
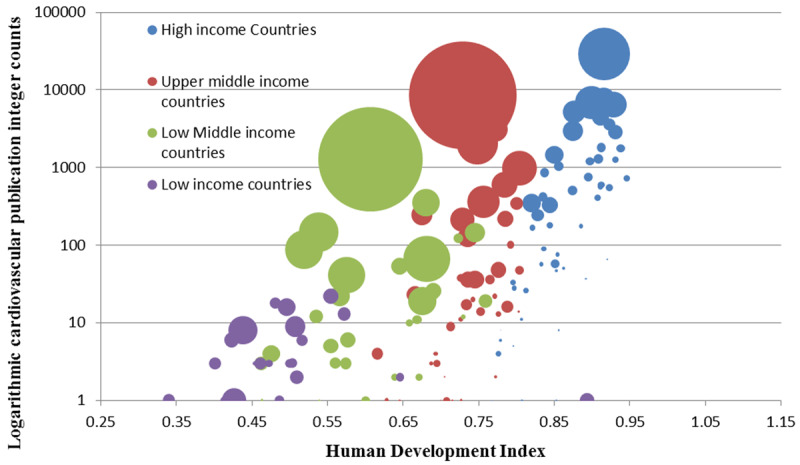
Logarithmic cardiovascular publication integer counts against Human Development index for the year 2013. Each bubble represents a country (n = 181). Size of the bubble represents the country population and color represents World Bank income group.

Cardiovascular research output had a direct relationship with HDI. High income countries had the highest number of publications and highest HDIs while low-income countries had the lowest HDIs and lowest publication numbers (Figure 5).

### Cardiovascular Disease Prevalence, DALY Losses, Death Rates and Research Output

Over 10 years cardiovascular disease prevalence rates declined for all income groups except for upper middle income countries (HI: p < 0.001, UMI: p = 0.066, LMI: p = 0.002, LI: p = 0.028). The time trends and relationship between disease prevalence and publication counts is shown in Appendix H.

High-income, upper-middle-income, lower-middle-income and low-income countries accounted for 8.1%, 32.4%, 48.7% and 10.8% of global CVD DALY losses respectively and 8.5%, 34.4%, 46.7% and 10.2% of deaths respectively.

**Figure 6:**
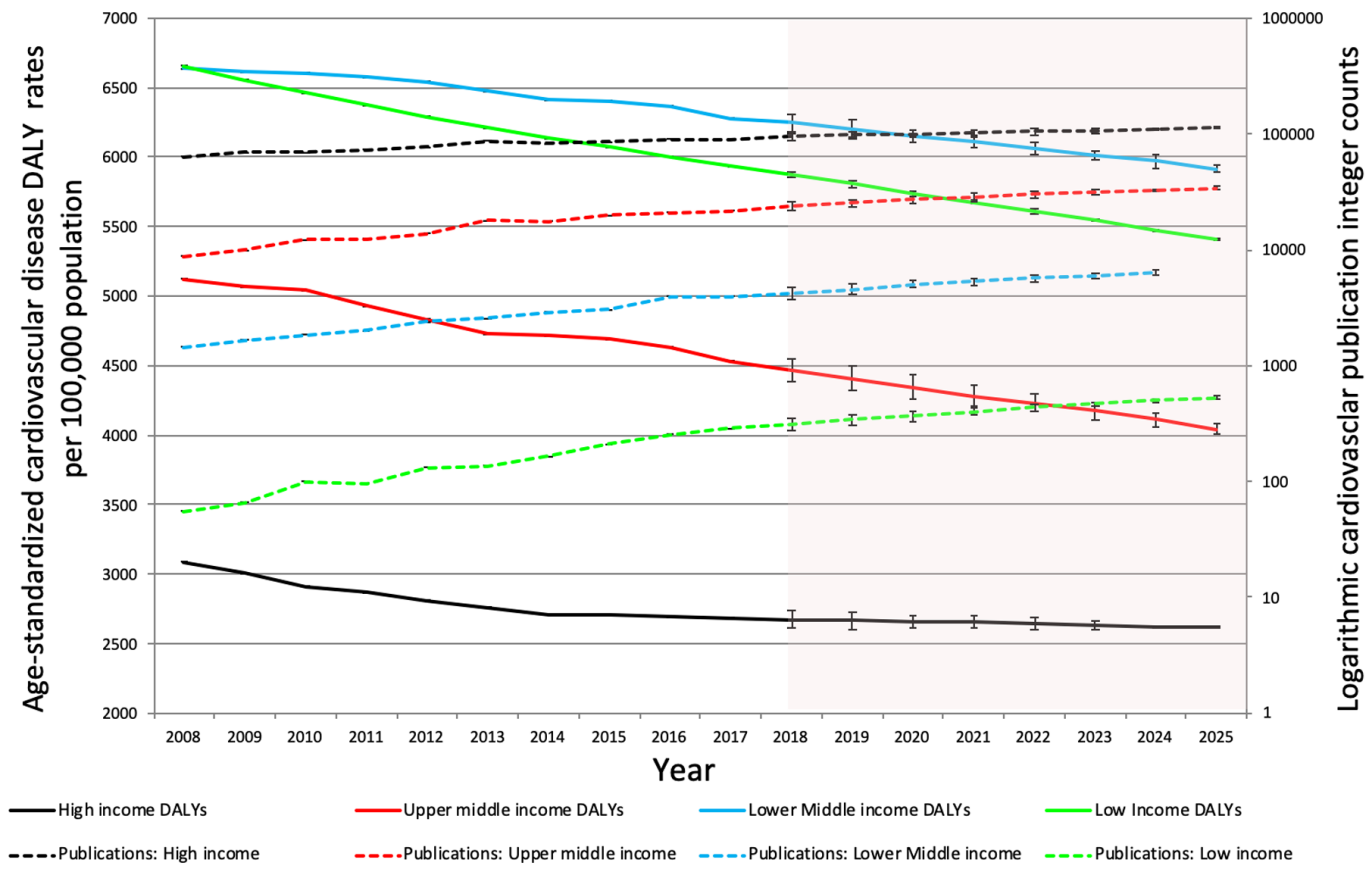
Trends in integer counts of cardiovascular publications and age-standardized DALY rates of cardiovascular disease in World Bank income groups (2008–2017). Shadowed portion represents forecasted values.

Cardiovascular disease morbidity, as reflected by DALY losses (HI: p = 0.001, UMI: p = 0.388, LMI: p = 0.892, LI: p = 0.001) and death rates (HI: p < 0.001, UMI: p = 0.002, LMI: p = 0.864, LI: p = 0.001) declined in all four income groups. While publication counts increased in all four income groups, the gap between cardiovascular morbidity and research output was widest for low income countries followed closely by lower middle income countries (Figures 6 and 8).

**Figure 7:**
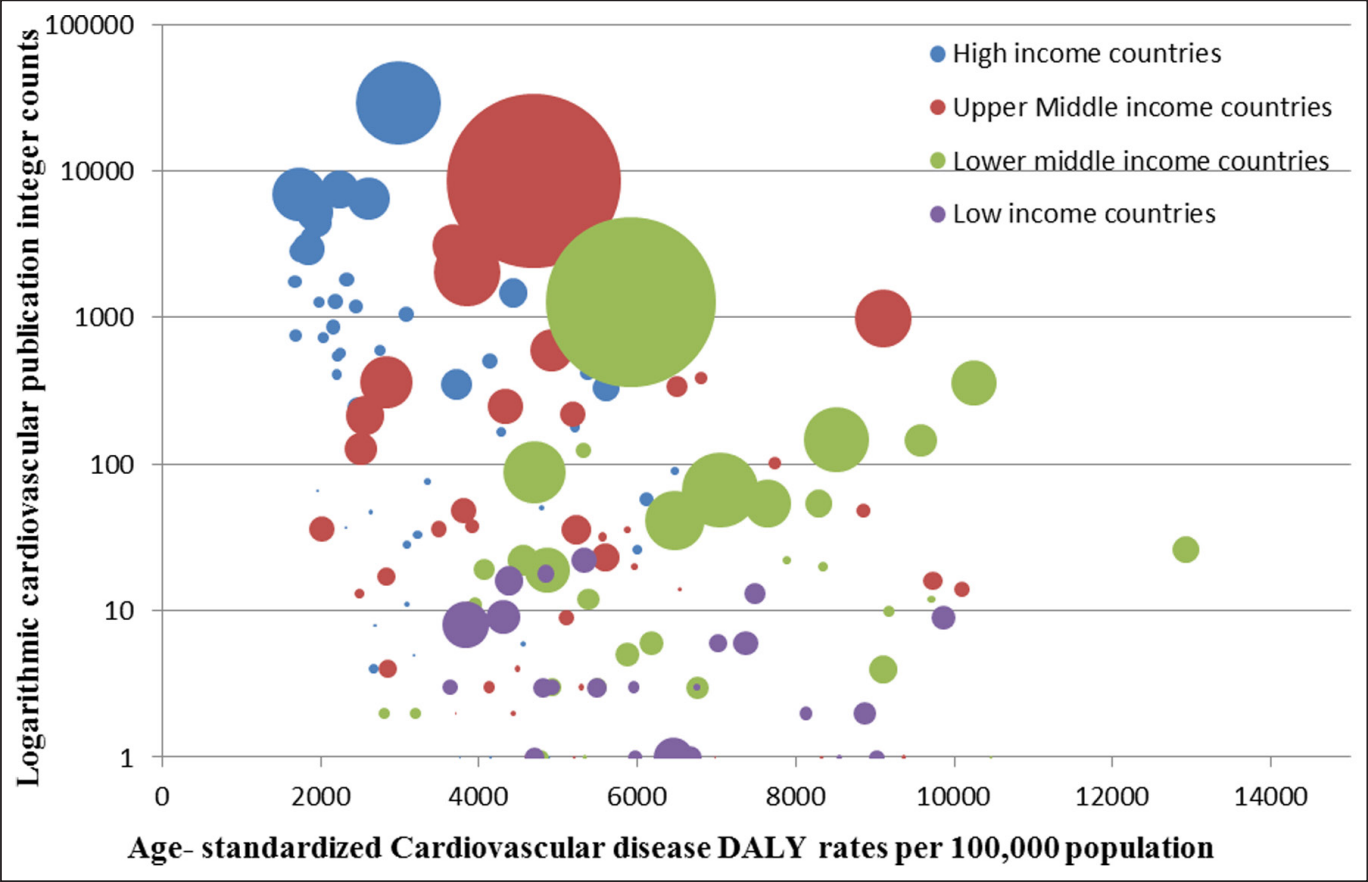
Logarithmic cardiovascular publication integer counts against age-standardized cardiovascular disease DALY rates per 100,000 population of for the year 2013. Each bubble represents a country (n = 181). Size of the bubble represents the country population and color represents World Bank income group.

Cardiovascular publication counts showed an inverse relationship with cardiovascular morbidity. High income countries have lowest death rates and DALY losses while the lower middle and low income groups showed the highest morbidity and lowest publication counts (Figures 7 and 9).

**Figure 8:**
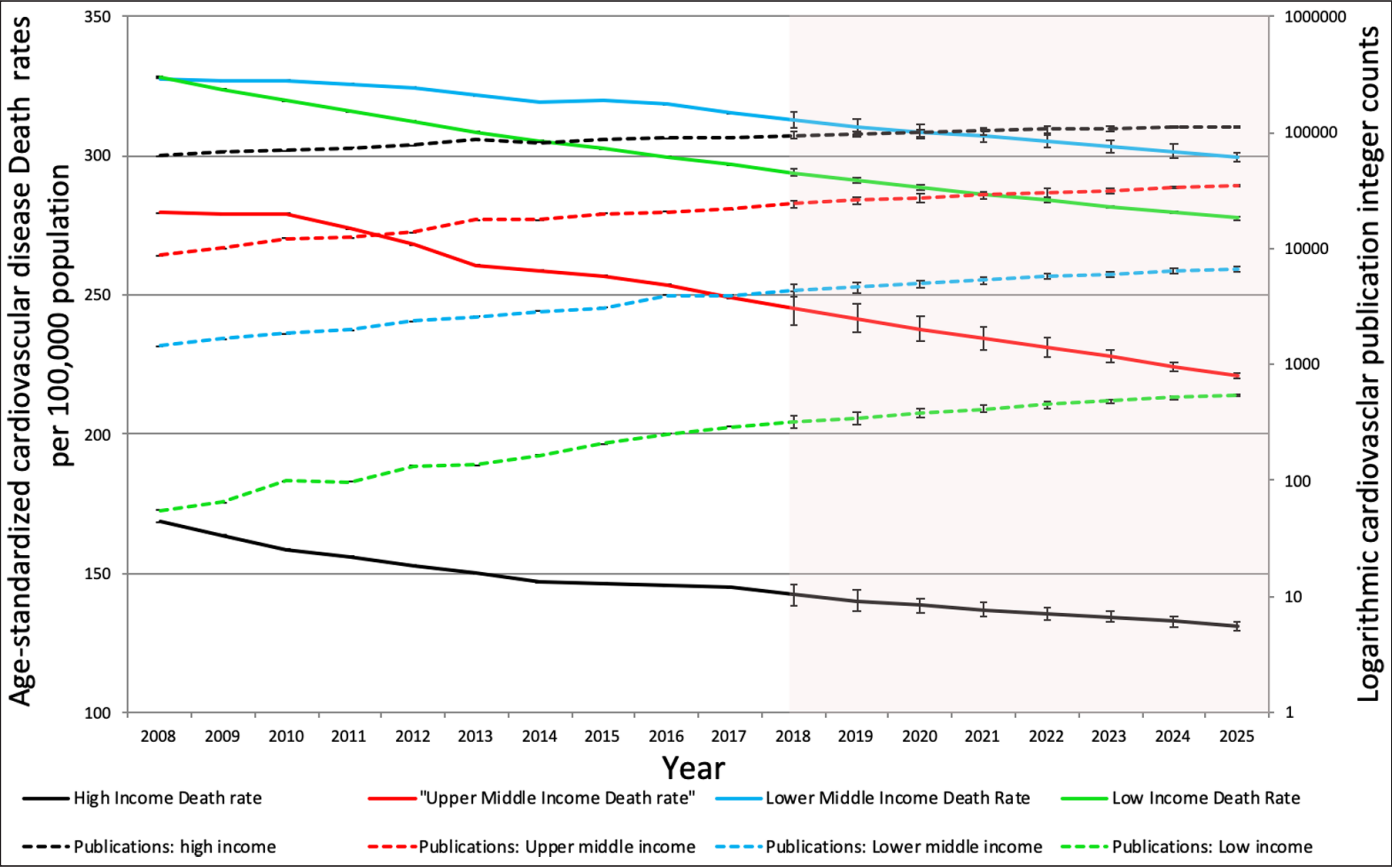
Trends in integer counts of cardiovascular publications and age-standardized death rates of cardiovascular disease in World Bank income groups (2008–2017). Shadowed portion represents forecasted values.

**Figure 9:**
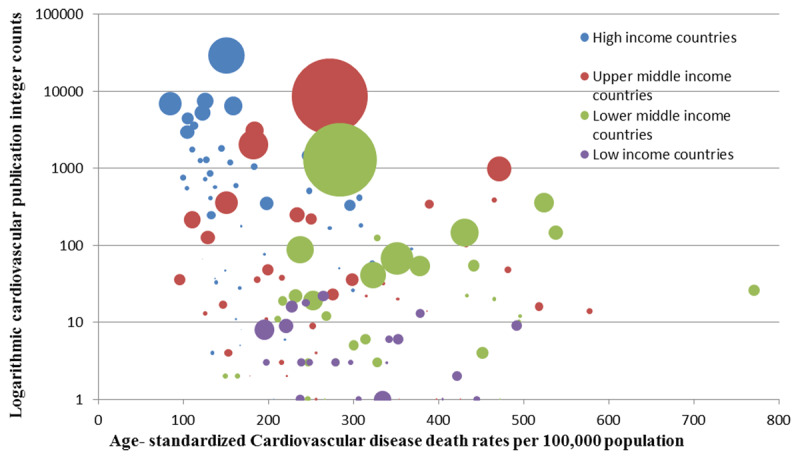
Logarithmic cardiovascular publication integer counts against age-standardized cardiovascular disease death rates per 100,000 population of for the year 2013. Each bubble represents a country (n = 181). Size of the bubble represents the country population and color represents World Bank income group.

### Significance of Changes in Prevalence, DALY and Death Rates

Table 2 shows that the decrease in the prevalence rates in 2017 compared to 2008 was insignificant for all four income groups. The high income group showed a significant decrease in death rates (*p* = 0.008) and DALY losses (*p* = 0.012) over the course of 10 years. Changes in DALY and death rates were insignificant in upper middle, lower middle and low income groups (Table 2).

**Table 2:** Percent changes in median cardiovascular disease prevalence, DALY and death rates in 2017 compared to 2008.

	2008	2017	% age decrease	p-value

Death Rate (per 100,000 population)				

HI	181 (142–225)	151 (116–227)	16.5%	0.008
UMI	271 (205–396)	248 (199–347)	8.4%	0.227
LMI	348 (259–485)	311 (239–434)	10.6%	0.185
LI	329 (261–397)	293 (251–356)	10.9%	0.226
**DALY (per 100,000 population)**				

HI	3195 (2519–4914)	2567 (1985–4030)	19.7%	0.012
UMI	5394 (4084–7076)	4378 (3741–6292)	18.8%	0.142
LMI	6917 (5214–9879)	6097 (4676–8435)	11.9%	0.151
LI	6451 (5245–8115)	5702 (4918–7149)	11.6%	0.251
**Prevalence (per 100,000 population)**				

HI	6484 ± 868.2	6370 ± 843.9	1.8%	0.497
UMI	6651 ± 822.3	6615 ± 802.7	0.5%	0.824
LMI	6260 ± 647.9	6230 ± 649.5	0.5%	0.832
LI	5918 (5624–6161)	5906 (5670–6252)	0.2%	0.773

**HI:** High income; **UMI:** Upper middle income; **LMI:** Low middle income; **LI:** Low income.

## Discussion

Cardiovascular publication output demonstrated a 43.4% increase from the time period 2008–2017; with a general increase noted among all income groups. An increase in international collaboration on cardiovascular publications was observed from 2010 to 2015. The United States contributed 27% of the global cardiovascular research output, the highest for a single country. However, highest publication rates (per 100,000 population) were observed for Iceland, Switzerland, Sweden, Norway and Austria. High- and upper-middle-income countries contributed 81.8% and 16.1%, to the global cardiovascular research output respectively. While low-middle to low-income countries house 50% of the world population, and burden 57% of global cardiovascular deaths and 59% of DALY losses, their contribution to CV research output remains dismal at only 2.8%. Among the four income groups, only high income countries have seen a significant decline in cardiovascular disease deaths and DALY losses. The differences are only expected to increase as shown by trend forecasting till the year 2025. Cardiovascular publication counts showed a direct relationship with HDI and an inverse relationship with DALY losses and death rates.

Evans et al. noted, that ‘research is a neglected key to the improvement of health, just as good health is an often-ignored key to development [37,38,39].’ The close link of cardiovascular research output and human development is evident through our work and consistent with findings from Huffman et al. [13] In comparison to a 36% increase in publication counts for the years 1998–2008, our study shows a 43% increase from 2008 to 2017. The steady increase in global cardiovascular research output parallels many others markers of continued improvement in several development indicators across the world [40].

The consequences of cardiovascular epidemics have been managed successfully in developed nations through comprehensive research informed strategies. Faced with a rising burden and high mortality from cardiovascular diseases, the National Institute of Health (NIH) since the 1940s heavily invested in research to understand the burden, risk factors and mechanisms of cardiovascular disease [41,42]. The Framingham study is an exemplar of this early investment; its learnings added to that of many others have contributed to a steady decline of cardiovascular mortality over time with global impact [43,44,45]. The increase in cardiovascular research output seen in our results coincides with a decline in CVD DALY and death rates in high, lower middle and low income countries (Figures 5 and 7). Countries with the highest publication rates in have cardiovascular disease health indicators among the best in world [46]. Research productivity and output in the form of research publications by a country may serve as a surrogate for awareness, advocacy and health sector investment to tackle disease burden.

Considering the role of research may play in impacting disease burden, a glaring disparity in the research output and disease burden persists. Low- and lower-middle-income countries account for 57% of CVD deaths yet contribute only 2.8% of global cardiovascular research output. This pattern has persisted across three decades. Prabhakaran et al. compared trends in cardiovascular publications in the four income groups from 1994–1995 and 2004–2005, found high, upper middle, lower middle and low income country contributions to global CV publications to be 82.4%, 7.1%, 6.6% and 3.9% respectively [25]. Mendis et al. showed similar trends [26].

While other factors such as population growth, lifestyle changes and rural to urban transition may have contributed to the decline in CVD burden, one reason behind reduction of CVD burden in the low and lower middle income group along with the high-income group reflects the global reach and possible transfer of knowledge generated from the bulk of research conducted in high-income countries to lower-middle- and low-income countries. Despite the benefit that low- and lower-middle-income countries may extract from ‘borrowing’ research conducted in high-income countries, the global distribution of cardiovascular disease burden raises the need for evidence to define contextual approaches to manage the NCD epidemic in low- and low-middle-income countries. The need for country and income specific research is three fold. Firstly, geographical and racial differences may affect disease biology and drug pharmacokinetics and dosages. For example, studies have shown that South Asians may have a genetic predisposition towards early and severe coronary artery disease. Similarly, many studies have shown variations in drug dosing based on racial ethnic differences on account of genetic polymorphisms in drug metabolism [47,48,49,50]. Secondly, translation and implementation of existing evidence is tricky and needs to be nuanced, contextualized for regions and informed by local data, numbers, geography, social-cultural, religious norms and most importantly resources [51]. Lastly, cardiovascular diseases affecting low and lower middle income countries predominantly may be abandoned as issues of interest for research on the global platform. An example is rheumatic heart disease (RHD); low- and lower-middle-income countries continue to struggle with a high burden of RHD but little research is dedicated to investigating prevention and treatment modalities.

A combination of global and national strategies is needed to reduce the disparity in research output and reduce the skewed distribution of cardiovascular disease burden across the world. While population health is not a monotonic function of health research spending, funding does impact research and ultimately generates evidence to reduce disease burden; the disparity in research output calls for a re-evaluation of both national and international health research funding patterns. The Prioritized Research Agenda for Non Communicable Diseases published by the WHO in 2011 suggested that 5% international official development and 2% national health expenditure should be dedicated to NCD research in low- and lower-middle-income countries [52]. For less economically developed countries, it may be more challenging for the national financial construct to accommodate health research spending since other national priorities including social and political change, infectious diseases, stimulation of economic growth and low literacy rates may be competing for limited resources and funding. The health sector itself has to deal with unique challenges including a shortage of health care professionals and a predilection of efforts to address NCD burden in urban areas, while neglecting underserved regions. Despite the obstacles, efforts should be made to dedicate funding to research from within existing health budgets.

‘Foreign aid’ packages and specific research funding organizations such as BMGF or the NIH are a source of international support to low and low middle income countries for health research spending. NCDs have contributed to a majority of mortality in low and lower middle income countries (67% in 2016) [53], little funding has been allocated to NCDs in low and lower middle income countries by global research funding agencies. In response to the 10/90 gap first highlighted by the commission on health research and development in 1990, tuberculosis, AIDS and malaria, together responsible for 12% of deaths in low-income countries, have recently been the prime targets of international donors [54,55,56].

To maximize the efficiency and lucrativeness of the financial investments in NCD research in low- and low-middle-income countries, funds need to be channeled towards identifying ‘solutions’ for public health implementation and health care delivery. Low- and lower-middle-income countries would particularly benefit from translational and implementation research which studies and proposes strategies to improve access to quality care and primary prevention. The National institutes of Health have launched a new Center for Translation Research and Implementation Science (CTRIS) which plans to focus on the principle of turning ‘discoveries into improved health.’ Extension of this approach to low- and lower-middle-income countries may be the best way forward with increasing research efforts. The need for implementation science is further evidenced in our results with the contribution of the United States to global cardiovascular research output; despite generating 27% of the global cardiovascular research output, the US does not have the best CVD health indicators in the world.

The increase in research efforts in low and lower middle income countries need to be accompanied with and in-part caused by an increase in local research capacity to ensure sustainability of progress. The role of foreign support in building research capacity may be in technical, scientific and financial support for training skilled and dedicated researchers, creating training institutions and setting up exchange programs with low- and lower-middle-income countries. National universities need to prioritize creation of research centers and invest in training researchers including epidemiologists and clinical scientists who can lead and sustain local research efforts. Another indirect way to build capacity and increase research output from low- and lower-middle-income countries is greater collaboration on research projects. Countries known to play a leadership role in fostering such ‘North-south’ collaborations include USA, UK, and France [27]. There may also be great potential in ‘South-South’ collaborations which is yet to be harnessed. Our results indicate an increase in international collaboration from 2010 to 2015.

### Strengths and Limitations

The strengths of our study include analyses of trends in cardiovascular publications over 10 years in 181 countries. The publications have been extracted using a rigorously developed and tested search filter with a precision and recall >0.9 developed through iterative amendments based on text mining technology.

We used integer counts, so that our results could be comparable to analyses from prior publications [13,25,26,27]. While integer counts may over estimate publications counts in contrast to fractional counts, this over estimation likely affects all countries and therefore ratios and comparisons between countries are possible [13]. The search filter was developed using literature in English but the application of the filter was not restricted by language. While the web of science is the largest bibliometric database, publications in journals not indexed in the WOS and those in other languages may have been neglected in this analysis. High percentage increases observed for some countries may be a consequence of an increase in the number of open access ‘predator journals’ during the study time period.

The seminal work in our paper presents quantitative analyses of global cardiovascular publications to study the disparities in research output between high income and low and lower middle income countries. For deeper insight into local research capacity, a qualitative analyses into the fractional contributions, first and corresponding authorships, citation impact of publications, rural and urban distribution of research output, health issues and journals of focus and institutional research output in low- and lower-middle-income countries needs to be conducted.

## Conclusions

Cardiovascular research output has grown over the past decade. High-income countries are the major contributors of global cardiovascular research output with the lowest age-standardized Disability Adjusted Life Years and death rates due to cardiovascular disease. Lower-middle- and low-income countries contribute to less than one tenth of the publication output despite having the highest age-standardized Disability Adjusted Life Years and death rates. There is a need to generate evidence for targeting unique pathways of cardiovascular disease and strategies to strengthen efforts for translational and implementation research. National health budgets and international funding support need to allocate funds to strengthen research capacity and promote local efforts to target and impact cardiovascular disease burden in low and lower middle income countries.

## Additional Files

The additional files for this article can be found as follows:

10.5334/gh.815.s1 Appendix A. Integer counts for cardiovascular publications for countries in each income group (2008–2017).

10.5334/gh.815.s2 Appendix B. Fractional contribution estimates (sample- 2010 & 2015).

10.5334/gh.815.s3 Appendix C. Age Standardized Prevalence rates for countries in each income group (2008–2017).

10.5334/gh.815.s4 Appendix D. Age Standardized DALYs for countries in each income group (2008–2017).

10.5334/gh.815.s5 Appendix E. Age Standardized Death rates for countries in each income group (2008–2017).

10.5334/gh.815.s6 Appendix F. Total Population for countries in each income group (2008–2017).

10.5334/gh.815.s7 Appendix G. Human Development Index for countries in each income group (2008–2017).

10.5334/gh.815.s8 Appendix H. Cardiovascular publications and prevalence rates: Trends (A) and relationship (B).

10.5334/gh.815.s9 Appendix I. List of International Classification of Diseases (ICD) codes mapped to the Global Burden of Disease cause list for causes of death.

## Figures and Tables

**Table:** 

NQQ:	Collected the data, conceived and designed the analysis, performed the analysis, visualized the data and wrote the manuscript.
SHM:	Collected the data, conceived and designed the analysis, performed the analysis, visualized the data and wrote the manuscript.
GSB:	Designed the analysis, reviewed and edited the manuscript.
WT:	Collected the data.
AA:	Reviewed and edited the manuscript.
AHM:	Wrote, reviewed and edited the manuscript.
JB:	Reviewed and edited the manuscript.
IN:	Reviewed and edited the manuscript.
SS:	Reviewed and edited the manuscript.
ZB:	Wrote, reviewed and edited the manuscript.
DM:	Wrote, Reviewed and edited the manuscript.
PSD:	Wrote, Reviewed and edited the manuscript.
ZS:	Conceived and designed the analysis, wrote and reviewed the manuscript, supervised the project.

## Author Information

Dr. Jhon Bartlett serves as Editor, Global Health Section, up-to-date and receives royalties as compensation.

Dr. M Imran Nisar is partially supported by National Institute of Health’s Fogarty International Center (grant number 1 D43 TW007585-01).
