# Supplementary material for: Disparities in Cardiovascular Research Output and Disease Outcomes among High-, Middle- and Low-Income Countries – An Analysis of Global Cardiovascular Publications over the Last Decade (2008–2017)
Source: Glob Heart. 2021 Jan 18;16(1):4. doi: 10.5334/gh.815 (PMC7845477; doi:10.5334/gh.815)
Supplement: Appendix A. — Integer counts for cardiovascular publications for countries in each income group (2008–2017). [file gh-16-1-815-s1.pdf]

Cardiovascular publications- High Income Countries (2008-2017) (Integer counts)

Country name	2008	2009	2010	2011	2012	2013	2014	2015	2016	2017
Andorra	0	0	0	0	0	0	0	0	0	1
Antigua and Barbuda	3	0	0	0	0	1	0	3	0	3
Argentina	460	285	316	303	307	349	335	359	382	352
Australia	1686	1901	1973	2175	2502	2853	3024	3041	3362	3225
Austria	743	899	933	986	912	1199	1170	1144	1238	1242
Bahrain	11	14	21	10	25	11	14	37	18	15
Barbados	3	5	3	7	1	5	6	6	7	5
Belgium	990	1057	1023	1042	1203	1283	1254	1320	1302	1370
Brunei	3	1	1	2	5	1	7	7	10	3
Canada	3024	3393	3517	3690	3886	4452	4174	4267	4590	4628
Chile	136	124	159	179	166	245	251	295	262	277
Croatia	107	199	166	150	171	167	168	192	198	163
Cyprus	17	24	29	32	32	47	47	64	69	76
Czech Republic	408	465	444	427	440	508	584	617	636	630
Denmark	707	909	931	988	1143	1266	1371	1403	1512	1648
Estonia	23	47	42	39	41	50	49	60	55	52
Finland	573	609	592	591	577	599	684	682	702	711
Germany	5135	5644	5589	5855	6112	6460	6147	6396	6486	6554
Greece	1180	1248	1233	1098	1044	1049	1149	1172	1156	1073
Hungary	316	326	390	366	360	417	507	494	464	470
Iceland	35	84	53	42	62	66	75	91	74	84
Ireland	285	302	460	497	512	571	618	583	662	677
Israel	636	659	627	682	640	752	738	769	849	760
Italy	3884	4286	4441	4502	4605	5242	4864	5392	5550	5485
Japan	5330	5630	5614	6051	6433	6936	6372	5985	6771	6135
Kuwait	49	47	37	38	36	33	31	71	40	40
Latvia	13	40	23	42	39	57	40	49	52	50
Lithuania	83	113	82	77	83	90	105	106	126	149
Luxembourg	16	5	20	26	32	37	52	35	49	52
Malta	2	1	2	8	10	8	13	19	20	26
Netherlands	2484	3050	2935	3184	3191	3569	3347	3481	3468	3524
New Zealand	342	335	366	384	379	409	429	522	465	447

Norway	538	622	657	635	727	728	783	834	801	788
Oman	14	21	26	31	49	26	21	47	33	45
Panama	2	1	4	9	1	4	7	5	6	11
Poland	1145	1342	1340	1437	1441	1466	1313	1567	1666	1629
Portugal	307	393	640	494	587	858	754	824	822	875
Qatar	21	19	30	38	68	76	68	141	120	127
Saudi Arabia	82	108	137	196	285	331	364	391	404	431
Seychelles	3	5	2	2	4	8	0	1	1	3
Singapore	285	285	342	382	401	550	496	558	650	663
Slovakia	150	175	144	129	166	180	189	176	209	191
Slovenia	109	118	111	135	115	176	156	169	178	178
Spain	1966	2088	2333	2401	2503	2950	3004	3248	3119	3291
Sweden	1210	1380	1306	1429	1489	1815	1778	1873	1912	2030
Switzerland	1229	1350	1467	1627	1637	1767	1705	1863	1988	2077
The Bahamas	0	0	1	0	1	1	0	1	0	1
Trinidad and Tobago	3	6	4	5	6	6	8	5	4	6
United Arab Emirates	32	46	63	69	98	58	83	103	112	141
United Kingdom	5268	5513	5641	6003	6470	7505	7029	7847	7715	7768
United States	22,028	23618	23105	24714	26109	29136	26445	27285	29479	29150
Uruguay	35	29	27	31	26	28	43	39	34	38
	63111	68821	69402	73240	77132	86401	81871	85639	89828	89370

Cardiovascular publications- Upper Middle Income Countries (2008-2017)

Country name	2008	2009	2010	2011	2012	2013	2014	2015	2016	2017
Albania	13	13	26	10	17	22	29	28	30	23
Algeria	22	40	37	31	33	36	39	34	49	50
Armenia	40	36	41	11	22	20	21	15	15	32
Azerbaijan	9	7	17	12	9	14	12	23	15	14
Belarus	26	23	33	34	27	48	59	61	58	58
Belize	1	0	0	0	0	2	0	0	0	0
Bosnia and Herzegovina	31	45	44	50	21	32	27	41	39	29
Botswana	1	1	1	4	2	4	4	0	1	7
Brazil	1507	1527	1677	1774	1743	2045	1872	1994	2067	2093
Bulgaria	80	85	67	66	81	101	93	126	137	99
China	2847	3856	5297	5276	6352	8536	9213	10566	11180	12282
Colombia	46	55	74	60	86	126	111	131	165	231
Costa Rica	8	9	11	9	1	13	18	13	18	22
Cuba	24	23	22	29	26	36	26	30	14	24
Dominica	2	0	0	1	0	0	0	2	1	6
Dominican Republic	0	4	0	1	3	9	5	9	2	3
Ecuador	2	5	10	3	6	17	15	22	30	29
Equatorial Guinea	0	0	0	0	0	0	1	0	0	0
Fiji	2	1	0	1	2	1	2	2	6	4
Gabon	4	0	0	1	3	3	2	2	5	3
Grenada	6	14	18	13	18	17	20	21	19	9
Guatemala	4	0	1	4	5	4	8	13	7	7
Guyana	2	1	0	0	0	1	1	1	2	2
Iran	380	440	447	639	655	599	573	637	757	780
Iraq	13	11	13	12	17	23	23	23	22	26
Jamaica	13	13	8	16	14	11	11	12	11	13
Jordan	16	16	25	33	27	38	34	60	62	66
Kazakhstan	0	6	3	6	4	16	24	57	47	39
Libya	5	2	4	2	3	1	6	1	4	2
Macedonia	23	29	34	26	15	36	33	36	45	37
Malaysia	87	95	128	160	206	218	232	275	323	422
Maldives	0	0	1	0	0	0	0	0	1	0

Marshall Islands	0	0	0	0	0	0	0	0	0	0
Mauritius	2	0	2	1	1	2	3	5	1	2
Mexico	203	223	242	227	267	360	334	355	398	436
Montenegro	4	8	6	9	2	14	18	26	27	15
Namibia	0	0	0	0	1	1	1	2	2	1
Paraguay	2	1	5	1	3	3	3	8	10	9
Peru	14	19	17	12	22	36	50	60	29	52
Romania	226	262	372	272	275	342	368	531	472	470
Russian Federation	852	887	958	942	964	986	1080	1306	1328	1418
Saint Lucia	0	0	0	0	0	0	0	0	0	0
Saint Vincent and the Grenadines	0	0	0	0	0	0	0	1	1	0
Samoa	0	0	0	1	0	1	0	0	0	4
Serbia	242	276	310	339	363	387	387	445	418	407
South Africa	178	195	177	188	238	248	285	302	375	338
Suriname	1	1	0	0	0	1	1	1	7	5
Thailand	132	203	182	187	225	215	257	243	301	328
Tonga	1	0	0	0	0	0	0	0	0	0
Turkey	1626	1489	1827	1840	1935	3104	2290	2453	2269	1927
Turkmenistan	0	1	0	0	1	0	0	3	0	1
Venezuela	54	49	67	32	27	48	49	42	31	31
	8751	9971	12204	12335	13722	17777	17640	20018	20801	21856

Cardiovascular publications- Lower Middle Income Countries (2008-2017)

Country name	2008	2009	2010	2011	2012	2013	2014	2015	2016	2017
Angola	1	2	0	1	2	5	5	5	5	8
Bangladesh	13	16	24	22	41	41	43	67	68	57
Bhutan	0	0	0	0	0	0	0	1	1	0
Bolivia	10	6	12	10	6	11	7	12	9	11
Cambodia	0	0	0	1	1	3	4	2	6	3
Cameroon	12	13	12	13	19	12	30	24	53	49
Cape Verde	0	0	0	0	0	0	0	0	0	2
Congo	2	1	1	2	1	0	1	4	6	7
Cote d'Ivoire	3	3	1	5	8	3	6	4	14	13
Djibouti	0	1	0	1	0	1	0	0	0	0
Egypt	142	216	269	271	314	356	383	464	515	596
El Salvador	0	1	1	0	0	2	0	1	0	3
Federated States of Micronesia	0	0	0	0	0	0	1	0	0	0
Georgia	30	39	25	30	12	20	28	20	22	24
Ghana	5	8	2	11	11	6	18	27	36	58
Honduras	0	0	1	0	1	1	2	2	1	5
India	758	793	850	973	1166	1286	1251	1231	1713	1437
Indonesia	48	54	50	54	70	67	228	162	314	416
Kenya	4	10	13	13	19	22	16	23	36	37
Kiribati	0	0	0	0	0	0	0	0	0	0
Kyrgyzstan	5	8	4	3	7	10	4	10	12	36
Laos	2	0	0	1	0	0	0	0	1	1
Lesotho	0	0	0	0	0	0	0	0	1	0
Mauritania	0	0	2	0	1	0	0	1	2	3
Moldova	16	3	16	17	12	22	26	13	11	19
Mongolia	2	3	5	4	4	12	11	14	18	19
Morocco	33	49	33	40	60	54	52	85	50	56
Myanmar	0	0	1	3	0	0	0	4	3	5
Nicaragua	0	0	1	2	1	2	1	1	0	4
Nigeria	71	65	66	68	103	88	100	97	134	139
Pakistan	61	87	118	139	149	147	189	183	216	254
Papua New Guinea	0	0	0	0	1	0	0	0	0	3

Philippines	23	25	43	38	51	54	73	71	69	81
Sao Tome and Principe	0	0	0	0	0	0	0	0	0	2
Solomon Islands	0	0	0	0	1	1	0	0	0	1
Sri Lanka	11	14	15	15	18	19	17	34	41	62
Sudan	3	5	4	3	4	4	9	10	26	25
Timor-Leste	0	0	0	0	0	0	0	0	0	0
Tunisia	57	92	102	124	124	124	137	128	158	147
Ukraine	99	99	123	89	101	146	173	242	234	221
Uzbekistan	22	18	23	17	28	26	35	58	79	88
Vanuatu	2	0	0	1	0	0	0	1	1	1
Vietnam	11	16	14	19	28	19	33	46	68	58
Zambia	0	2	1	0	2	3	1	5	4	9
	1446	1649	1832	1990	2366	2567	2884	3052	3927	3960

Cardiovascular publications- Low Income Countries (2008-2017)

Country name	2008	2009	2010	2011	2012	2013	2014	2015	2016	2017
Afghanistan	1	0	2	1	1	0	3	3	2	3
Benin	2	1	3	3	5	3	4	3	8	12
Burkina Faso	1	0	3	2	1	3	4	7	8	7
Burundi	0	0	0	0	1	1	0	0	2	0
Central African Republic	0	1	3	0	1	0	1	1	3	1
Chad	0	0	0	0	0	0	0	1	1	0
Comoros	1	0	0	0	0	0	0	0	0	0
Democratic Republic of the Congo	3	1	0	1	3	1	5	5	7	9
Eritrea	0	0	1	2	0	1	2	0	0	1
Ethiopia	2	3	6	4	6	8	13	19	21	43
Guinea	0	0	1	0	0	0	1	1	6	5
Guinea-Bissau	0	0	0	0	0	1	0	0	0	1
Haiti	1	1	3	1	1	1	4	2	3	3
Liberia	0	0	0	0	0	0	0	0	0	0
Madagascar	2	0	0	4	1	2	3	2	1	1
Malawi	3	1	3	4	8	3	4	11	14	11
Mali	0	1	1	0	2	0	0	1	5	1
Mozambique	5	4	9	8	11	6	13	12	24	19
Nepal	9	17	18	19	15	22	28	21	36	33
Niger	0	0	0	2	1	1	1	2	4	6
North Korea	1	0	0	0	0	1	0	1	2	2
Rwanda	1	4	5	3	2	3	4	7	4	7
Senegal	2	4	4	8	11	18	12	13	17	20
Sierra Leone	0	1	1	0	0	0	0	0	0	2
South Sudan	0	0	0	0	0	0	0	0	1	0
Syria	2	3	4	8	8	13	11	8	7	9
Tajikistan	1	1	0	0	1	2	0	1	0	0
Tanzania	6	5	7	11	11	9	13	17	23	32
The Gambia	1	3	2	1	0	3	1	1	0	2
Togo	1	0	1	3	2	3	1	6	6	4
Uganda	3	8	11	4	11	16	19	31	32	35
Yemen	6	6	11	7	29	9	11	23	6	12

Zimbabwe	1	0	0	0	0	6	6	9	10	7
	55	65	99	96	132	136	164	208	253	288
