# Supplementary material for: Disparities in Cardiovascular Research Output and Disease Outcomes among High-, Middle- and Low-Income Countries – An Analysis of Global Cardiovascular Publications over the Last Decade (2008–2017)
Source: Glob Heart. 2021 Jan 18;16(1):4. doi: 10.5334/gh.815 (PMC7845477; doi:10.5334/gh.815)
Supplement: Appendix B. — Fractional contribution estimates (sample- 2010 & 2015). [file gh-16-1-815-s2.pdf]

2010				2015			
**Country Name**	**Fractional count**	**Integer count**	**Fractional contribution estimate**	**Country Name**	**Fractional count**	**Integer count**	**Fractional contribution estimate**
**Total**	384.0163	433	0.886874	Total	384.4293	459	0.837537
**Albania**	1	1	1	**Albania**	1	1	1
**Australia**	12.54286	14	0.895918	**Argentina**	4.105263	5	0.821053
**Austria**	4.25	5	0.85	**Australia**	6.761364	9	0.751263
**Bolivia**	0.166667	1	0.166667	**Austria**	6	6	1
**Brazil**	6.333333	7	0.904762	**Belarus**	1	1	1
**Cameroon**	0.2	1	0.2	**Belgium**	2.153846	3	0.717949
**Canada**	19.34444	21	0.921164	**Bolivia**	0.5	1	0.5
**Chile**	1	1	1	**Brazil**	4.24906	7	0.607009
**China**	34.84949	37	0.941878	**Canada**	18.77598	25	0.751039
**Croatia**	0.75	1	0.75	**China**	46.02976	48	0.958953
**Czech Republic**	1	1	1	**Croatia**	0.666667	1	0.666667
**Denmark**	2.142857	3	0.714286	**Czech Republic**	0.125	1	0.125
**Egypt**	1	1	1	**Denmark**	4.125	5	0.825
**Finland**	2	2	1	**Egypt**	4	4	1
**Germany**	20.94643	23	0.910714	**Estonia**	0.375	1	0.375
**Greece**	7	7	1	**Finland**	1.215909	3	0.405303
**Hungary**	2	2	1	**Georgia**	1	1	1
**India**	5	5	1	**Germany**	13.90397	17	0.81788
**Iran**	3.595238	4	0.89881	**Ghana**	0.083333	1	0.083333
**Ireland**	4	4	1	**Greece**	4.833333	6	0.805556
**Italy**	24.24508	26	0.932503	**Hungary**	1	1	1
**Japan**	16	16	1	**India**	4.875	6	0.8125
**Jordan**	1	1	1	**Iran**	3.714286	4	0.928571
**Latvia**	1	1	1	**Ireland**	2.5	4	0.625
**Malaysia**	1	1	1	**Israel**	2	2	1
**Mozambique**	0.1	1	0.1	**Italy**	17.43558	20	0.871779
**Netherlands**	15.85186	20	0.792593	**Jamaica**	0.166667	1	0.166667
**Nigeria**	0.2	1	0.2	**Japan**	33.14286	34	0.97479
**Norway**	5	5	1	**Kuwait**	2	2	1
**Philippines**	1.857143	2	0.928571	**Lithuania**	1	1	1
**Poland**	4.580556	6	0.763426	**Mexico**	2.333333	3	0.777778
**Portugal**	4	4	1	**Montenegro**	0.375	1	0.375
**Romania**	3	3	1	**Netherlands**	14.67454	18	0.815252
**Russian Federation**	7	7	1	**New Zealand**	0.117647	1	0.117647
**Serbia**	2	2	1	**Norway**	4.564286	6	0.760714

**Singapore**	0.833333	1	0.833333	**Pakistan**	1	1	1
**Slovakia**	2	2	1	**Peru**	1	1	1
**South Africa**	0.2	1	0.2	**Poland**	6.9	8	0.8625
**Spain**	15.12266	17	0.889568	**Portugal**	4.907895	6	0.817982
**Sudan**	0.1	1	0.1	**Qatar**	1.125	2	0.5625
**Sweden**	4.111111	5	0.822222	**Romania**	0.8	1	0.8
**Switzerland**	2.854762	5	0.570952	**Russian Federation**	3.914286	6	0.652381
**Turkey**	6.555556	7	0.936508	**Saudi Arabia**	0.5	1	0.5
**Uganda**	0.1	1	0.1	**Serbia**	1.625	2	0.8125
**Ukraine**	1.666667	2	0.833333	**Singapore**	1.117647	2	0.558824
**United Arab Emirates**	1	1	1	**Slovakia**	0.875	1	0.875
**United Kingdom**	20.52035	27	0.760013	**South Africa**	0.303114	3	0.101038
**United States**	112.9959	126	0.896793	**Spain**	6.897436	8	0.862179
				**Sweden**	3.995581	7	0.570797
				**Switzerland**	5.541667	8	0.692708
				**The Bahamas**	1	1	1
				**Tunisia**	1	1	1
				**Turkey**	9.833333	10	0.983333
				**United Kingdom**	21.56164	26	0.829294
				**United States**	97.72902	112	0.872581
				**Uruguay**	1	1	1
				**Uzbekistan**	1	1	1

Fractional counts, integer counts and fractional contribution estimates for a sample of 383 articles each from the years 2010 and 2015. Higher fractional contribution estimates reflect a lower degree of international collaboration.
