# Supplementary material for: Disparities in Cardiovascular Research Output and Disease Outcomes among High-, Middle- and Low-Income Countries – An Analysis of Global Cardiovascular Publications over the Last Decade (2008–2017)
Source: Glob Heart. 2021 Jan 18;16(1):4. doi: 10.5334/gh.815 (PMC7845477; doi:10.5334/gh.815)
Supplement: Appendix C. — Age Standardized Prevalence rates for countries in each income group (2008–2017). [file gh-16-1-815-s3.pdf]

Age Standardized Prevalence rates- High Income countries (2008-2017)

Country Name	2008	2009	2010	2011	2012	2013	2014	2015	2016	2017
Andorra	6321.428	6284.957	6263.113	6234.25	6215.724	6207.295	6206.339	6206.142	6210.602	6218.413
Antigua and Barbuda	6773.689	6764.244	6755.018	6744.45	6733.554	6720.484	6707.523	6690.243	6673.793	6656.241
Argentina	6069.336	6047.143	6032.915	6023.126	6018.628	6015.386	6015.088	6018.149	6026.085	6034.071
Australia	5076.694	5057.63	5041.988	5027.417	5013.67	4999.235	4984.761	4971.323	4958.345	4946.159
Austria	6781.935	6782.155	6776.037	6760.394	6737.23	6710.682	6677.551	6637.691	6591.155	6545.015
Bahrain	6718.662	6705.267	6696.164	6694.272	6692.506	6691.376	6694.269	6698.815	6704.196	6713.185
Barbados	6822.593	6819.285	6816.528	6814.602	6812.634	6810.776	6807.107	6807.674	6806.288	6804.878
Belgium	7350.084	7326.746	7301.942	7271.092	7231.069	7182.734	7125.089	7061.786	6992.843	6916.48
Brunei	5491.805	5476.221	5457.587	5436.592	5410.632	5381.079	5350.01	5310.933	5274.016	5235.048
Canada	6833.782	6800.386	6783.237	6774.698	6770.04	6766.084	6766.833	6767.85	6772.842	6781.297
Chile	5145.728	5139.778	5137.554	5135.948	5138.911	5143.838	5150.483	5160.163	5170.478	5184.765
Croatia	7002.215	6992.618	6984.736	6977.323	6976.286	6978.726	6984.817	6991.569	7002.892	7014.515
Cyprus	5744.95	5717.148	5694.476	5671.836	5650.294	5627.518	5608.226	5588.09	5568.775	5551.562
Czech Republic	8469.415	8514.448	8527.04	8514.683	8502.816	8490.535	8481.102	8472.446	8463.903	8457.221
Denmark	6904.59	6837.479	6779.304	6728.385	6673.219	6616.79	6562.358	6507.374	6454.454	6402.488
Estonia	6481.258	6452.626	6428.12	6404.203	6381.74	6356.115	6331.588	6306.859	6281.227	6256.38
Finland	6523.975	6502.496	6483.102	6460.587	6430.223	6397.84	6360.084	6320.185	6278.363	6233.681
Germany	6668.213	6645.517	6633.445	6629.328	6625.874	6622.065	6619.81	6616.718	6612.157	6607.237
Greece	5887.445	5888.606	5883.439	5880.623	5877.65	5874.81	5873.705	5871.948	5870.515	5867.45
Hungary	7646.162	7671.285	7673.142	7656.641	7637.167	7616.471	7595.02	7569.279	7541.271	7512.764
Iceland	6257.874	6223.671	6200.645	6182.336	6162.542	6143.494	6121.634	6102.91	6085.464	6068.739
Ireland	6119.984	6088.131	6062.985	6044.285	6026.351	6011.216	5996.777	5986.938	5977.351	5972.045
Israel	5673.605	5642.668	5621.311	5604.251	5589.021	5572.204	5559.025	5545.525	5533.571	5523.235
Italy	5971.275	5990.026	5985.165	5962.537	5936.41	5901.185	5859.333	5807.399	5750.126	5683.064
Japan	5445.524	5439.565	5430.574	5418.709	5404.669	5388.532	5370.081	5349.325	5326.969	5302.597
Kuwait	7263.202	7261.435	7256.019	7245.188	7228.234	7207.468	7180.971	7147.17	7112.009	7071.558
Latvia	6237.94	6254.818	6264.893	6268.104	6269.868	6268.449	6264.933	6258.374	6246.267	6232.247
Lithuania	6764.981	6799.086	6819.231	6832.368	6844.76	6857.321	6867.217	6879.24	6888.308	6892.778
Luxembourg	8590.416	8472.878	8379.142	8306.971	8237.569	8169.242	8107.018	8052.479	8000.581	7955.057
Malta	5752.053	5725.627	5707.479	5695.449	5689.536	5690.955	5699.61	5714.104	5733.489	5757.586
Netherlands	6321.891	6297.013	6280.251	6268.692	6256.884	6242.659	6227.009	6209.753	6195.323	6178.177
New Zealand	5056.891	5051.435	5044.973	5036.556	5027.515	5016.889	5002.961	4987.751	4969.603	4950.658

Norway	5259.957	5259.209	5259.471	5260.28	5260.894	5260.176	5259.085	5257.619	5255.639	5253.583
Oman	8079.659	8095.863	8111.917	8131.303	8142.536	8132.622	8117.2	8095.53	8074.74	8044.954
Panama	5868.333	5861.229	5856.066	5856.62	5859.152	5862.405	5867.449	5877.256	5885.434	5896.013
Poland	7211.33	7235.914	7247.975	7249.59	7248.591	7248.39	7247.007	7246.826	7244.841	7239.072
Portugal	5847.501	5758.491	5713.507	5703.122	5690.211	5678.79	5669.7	5661.571	5655.527	5651.3
Qatar	7611.414	7553.849	7510.167	7477.4	7435.19	7387.123	7330.842	7267.936	7202.557	7137.34
Saudi Arabia	7040.141	7059.789	7074.405	7086.81	7101.104	7117.412	7133.4	7152.263	7171.189	7191.403
Seychelles	6316.651	6300.839	6288.502	6276.522	6265.019	6254.225	6245.06	6236.287	6229.821	6222.87
Singapore	5339.045	5336.245	5331.288	5321.535	5307.25	5290.133	5269.378	5246.004	5218.858	5191.751
Slovakia	8190.594	8247.005	8264.035	8248.802	8228.278	8204.284	8174.059	8134.881	8083.139	8019.258
Slovenia	7041.135	7076.681	7084.974	7074.767	7065.332	7054.523	7044.684	7030.747	7014.858	6997.126
Spain	5619.035	5586.025	5565.171	5553.086	5543.925	5537.402	5531.596	5528.372	5530.131	5532.881
Sweden	6930.861	6900.552	6878.963	6866.465	6848.962	6831.318	6812.885	6793.539	6776.726	6762.51
Switzerland	5451.553	5446.104	5444.282	5451.137	5462.831	5478.087	5496.783	5519.585	5547.312	5579.281
The Bahamas	6196.924	6191.436	6186.709	6184.618	6183.137	6181.084	6179.761	6179.823	6178.123	6179.206
Trinidad and Tobago	6922.264	6921.928	6920.543	6916.717	6918.054	6921.534	6925.523	6931.632	6941.604	6950.718
United Arab Emirates	6824.674	6816.54	6823.452	6834.236	6844.416	6857.135	6869.417	6885.457	6902.911	6917.231
United Kingdom	6366.952	6355.234	6342.747	6328.195	6311.672	6293.083	6272.528	6250.041	6225.816	6199.858
United States	7383.229	7389.988	7386.385	7374.577	7361.656	7347.756	7332.094	7315.362	7296.357	7275.8
Uruguay	5506.761	5482.443	5466.576	5457.257	5448.268	5442.884	5439.803	5439.614	5441.745	5449.072

Age Standardized Prevalence rates- Upper middle income countries (2008-2017)

Country Name	2008	2009	2010	2011	2012	2013	2014	2015	2016	2017
Albania	8296.967	8288.969	8282.524	8278.839	8277.75	8272.135	8265.967	8263.655	8262.087	8261.242
Algeria	7076.311	7086.684	7093.97	7096.887	7098.577	7102.313	7104.324	7105.97	7105.296	7104.67
Armenia	6962.394	6964.74	6965.965	6956.914	6944.603	6926.194	6904.395	6877.18	6844.458	6808.093
Azerbaijan	6946.502	6987.368	7004.27	7004.798	6998.239	6983.783	6963.081	6936.659	6903.93	6861.531
Belarus	6760.978	6803.358	6818.226	6810.278	6791.943	6762.934	6721.049	6666.567	6598.992	6519.256
Belize	6675.521	6665.57	6658.48	6647.453	6638.234	6628.128	6619.772	6611.43	6603.623	6592.073
Bosnia and Herzegovina	8117.009	8130.505	8137.023	8138.178	8137.988	8135.629	8130.695	8124.494	8114.412	8102.041
Botswana	6075.517	6082.572	6091.116	6100.692	6112.72	6127.44	6142.529	6158.26	6174.296	6192.778
Brazil	6032.457	6027.294	6022.583	6018.48	6016.783	6016.435	6017.009	6019.058	6021.943	6024.971
Bulgaria	8890.206	8913.287	8921.93	8920.262	8910.276	8895.567	8872.514	8844.703	8808.289	8765.719
China	5364.365	5373.532	5384.046	5399.732	5423.242	5454.365	5491.162	5533.717	5580.518	5631.583
Colombia	5256.888	5247.653	5239.611	5233.766	5227.243	5222.699	5218.785	5216.81	5215.112	5215.046
Costa Rica	6030.808	6021.972	6013.264	6009.399	6004.821	6002.599	6000.649	5999.651	5998.481	5998.409
Cuba	5796.449	5804.007	5803.224	5799.203	5792.38	5783.733	5774.349	5763.15	5751.272	5734.778
Dominica	6332.659	6327.743	6322.44	6320.161	6316.821	6314.497	6313.882	6311.942	6309.342	6309.539
Dominican Republic	6220.419	6208.269	6199.259	6193.329	6186.325	6182.829	6175.918	6174.398	6171.319	6170.693
Ecuador	5506.949	5509.369	5512.977	5515.391	5517.626	5520.496	5522.19	5524.263	5527.044	5527.312
Equatorial Guinea	5527.028	5517.733	5512.832	5508.595	5506.036	5504.492	5504.321	5506.83	5509.559	5516.213
Fiji	7621.352	7609.766	7600.663	7591.047	7585.449	7578.279	7572.668	7568.537	7565.715	7565.001
Gabon	5917.498	5913.758	5910.498	5906.1	5899.725	5892.803	5884.212	5875.52	5865.123	5855.101
Grenada	6668.592	6670.526	6673.856	6675.38	6678.855	6681.208	6684.45	6687.164	6690.553	6695.697
Guatemala	5835.897	5841.316	5847.434	5854.464	5863.382	5874.013	5887.33	5904.236	5921.678	5941.437
Guyana	6586.037	6574.575	6562.977	6556.081	6546.303	6537.331	6531.531	6526.229	6522.418	6518.568
Iran	6662.766	6661.74	6662.208	6664.174	6663.443	6661.138	6657.458	6652.794	6648.468	6644.092
Iraq	8368.923	8353.06	8341.646	8337.878	8342.891	8356.289	8373.773	8398.798	8424.176	8455.905
Jamaica	6347.686	6360.532	6368.183	6376.236	6387.439	6405.162	6422.929	6447.251	6472.638	6499.203
Jordan	7222.78	7224.435	7222.206	7215.878	7209.412	7197.473	7182.734	7166.663	7148.367	7129.453
Kazakhstan	6927.297	6928.973	6924.723	6899.709	6851.816	6781.711	6695.754	6591.808	6471.764	6340.535
Libya	7317.308	7349.333	7371.422	7384.467	7397.743	7405.435	7412.304	7413.719	7417.101	7414.452
Macedonia	7315.25	7311.316	7307.056	7299.57	7291.331	7283.993	7273.631	7263.923	7249.998	7236.713
Malaysia	7375.643	7421.585	7444.036	7449.674	7455.024	7460.115	7461.98	7460.181	7456.312	7450.884
Maldives	5744.659	5720.308	5700.941	5684.193	5670.076	5655.855	5642.797	5632.378	5621.644	5615.619

Marshall Islands	7132.816	7128.22	7122.569	7116.257	7107.925	7096.986	7082.345	7065.623	7048.966	7027.365
Mauritius	6682.142	6662.289	6646.109	6636.198	6623.845	6612.103	6598.293	6584.922	6573.195	6560.565
Mexico	6062.149	6054.269	6051.31	6053.451	6059.308	6070.379	6084.632	6102.889	6124.966	6150.865
Montenegro	7349.116	7343.082	7333.78	7328.724	7326.164	7323.979	7323.736	7327.205	7329.207	7333.665
Namibia	5975.998	5958.593	5945.137	5931.289	5918.88	5905.948	5893.8	5882.532	5870.86	5861.351
Paraguay	6650.668	6642.29	6630.319	6621.73	6614.209	6603.588	6594.888	6585.017	6576.537	6567.211
Peru	5551.773	5560.179	5569.785	5577.609	5587.839	5597.291	5610.18	5621.774	5637.021	5651.762
Romania	7183.169	7182.295	7174.631	7162.008	7148.501	7133.375	7116.254	7097.868	7075.905	7052.479
Russian Federation	6249.516	6265.815	6280.031	6294.204	6309.645	6326.076	6343.361	6360.935	6379.316	6398.925
Saint Lucia	6722.732	6708.77	6698.908	6691.94	6683.085	6674.613	6664.665	6653.029	6644.57	6635.405
Saint Vincent and the Grenadines	6894.084	6893.105	6888.713	6885.138	6880.328	6876.035	6871.113	6863.944	6858.476	6853.271
Samoa	7223.466	7218.89	7212.692	7208.621	7206.08	7204.236	7204.473	7204.653	7203.811	7202.885
Serbia	7278.256	7277.737	7271.333	7255.163	7230.596	7196.824	7155.152	7107.119	7051.901	6991.897
South Africa	6211.448	6203.032	6193.719	6182.128	6167.664	6150.042	6130.813	6109.34	6085.391	6059.287
Suriname	6496.053	6504.696	6513.706	6522.516	6532.504	6542.55	6551.721	6562.911	6575.013	6587.416
Thailand	6429.244	6409.555	6398.217	6392.169	6387.677	6387.259	6390.65	6397.143	6405.905	6420.71
Tonga	7771.999	7767.105	7765.372	7761.567	7761.421	7762.337	7768.056	7773.024	7779.144	7785.939
Turkey	6712.978	6688.184	6669.63	6657.458	6647.55	6637.804	6632.719	6631.168	6632.135	6635.768
Turkmenistan	6477.902	6482.918	6486.978	6488.395	6489.211	6487.708	6485.232	6480.332	6471.235	6461.439
Venezuela	5036.356	5034.037	5033.137	5034.342	5038.768	5044.907	5054.284	5062.65	5073.744	5087.219

Age Standardized Prevalence rates- Lower middle Income countries (2008-2017)

Country Name	2008	2009	2010	2011	2012	2013	2014	2015	2016	2017
Angola	5653.157	5660.875	5664.715	5665.099	5662.071	5659.263	5653.646	5645.527	5637.108	5628.759
Bangladesh	6122.398	6158.976	6186.502	6206.76	6227.629	6245.536	6263.34	6276.915	6288.064	6296.294
Bhutan	5462.703	5467.767	5471.147	5475.379	5479.738	5484.097	5488.964	5495.351	5500.545	5506.811
Bolivia	4888.743	4903.566	4915.556	4926.741	4943.41	4963.195	4985.699	5008.571	5032.878	5058.554
Cambodia	5638.46	5628.874	5621.319	5611.634	5601.168	5591.89	5582.627	5569.191	5557.954	5546.32
Cameroon	5855.263	5851.635	5843.558	5833.941	5821.153	5803.66	5785.456	5763.081	5737.654	5712.38
Cape Verde	6659.099	6655.734	6649.953	6644.824	6641.049	6636.909	6633.733	6630.407	6628.101	6625.589
Congo	5834.78	5840.659	5838.149	5832.06	5821.334	5813.969	5801.483	5787.435	5772.838	5756.164
Cote d'Ivoire	6346.705	6333.83	6321.798	6307.425	6293.998	6278.214	6262.604	6244.934	6225.221	6205.782
Djibouti	6031.069	6046.927	6055.902	6060.718	6067.117	6071.347	6071.84	6073.818	6070.244	6067.924
Egypt	7684.649	7724.221	7747.216	7757.06	7765.685	7769.89	7771.683	7770.541	7763.233	7751.845
El Salvador	6136.388	6139.81	6143.183	6147.916	6156.608	6166.827	6180.813	6196.15	6213.254	6227.339
Federated States of Micronesia	6967.005	6967.311	6968.686	6967.25	6967.171	6968.433	6971.175	6974.854	6978.365	6983.47
Georgia	6907.538	6937.519	6957.066	6972.468	6989.78	7010.879	7031.659	7056.661	7082.525	7106.411
Ghana	6259.774	6258.767	6254.943	6247.067	6236.847	6223.304	6208.02	6186.701	6166.347	6141.372
Honduras	5880.481	5880.341	5880.149	5880.479	5882.818	5883.129	5885.529	5886.582	5890.125	5890.918
India	5228.651	5224.479	5220.204	5216.875	5215.119	5214.034	5213.659	5214.005	5214.094	5214.161
Indonesia	6038.655	6047.038	6052.053	6053.191	6051.983	6048.141	6042.089	6032.882	6022.942	6011.184
Kenya	5985.309	5974.316	5965.83	5959.492	5952.589	5945.666	5939.522	5934.313	5930.013	5926.915
Kiribati	7396.226	7385.091	7375.353	7364.265	7359.074	7354.293	7351.483	7347.639	7348.007	7346.368
Kyrgyzstan	6184.881	6182.372	6176.097	6164.784	6143.729	6119.853	6092.742	6056.783	6020.548	5977.777
Laos	6273.307	6265.884	6254.451	6236.868	6215.353	6190.146	6159.565	6128.503	6090.939	6049.967
Lesotho	5982.736	6002.574	6014.906	6023.254	6029.838	6034.939	6038.873	6041.847	6041.652	6040.686
Mauritania	6147.971	6147.847	6147.148	6144.198	6142.404	6139.242	6140.026	6137.051	6134.386	6129.853
Moldova	5934.214	5946.679	5956.935	5962.909	5968.986	5972.691	5976.65	5978.55	5977.493	5976.187
Mongolia	6811.108	6809.551	6806.869	6804.708	6800.443	6795.3	6790.753	6780.351	6770.45	6760.113
Morocco	7693.097	7694.466	7691.703	7685.319	7676.62	7665.282	7653.446	7636.542	7620.933	7602.36
Myanmar	5534.708	5528.093	5517.886	5507.503	5497.829	5486.79	5477.802	5467.124	5455.291	5442.518
Nicaragua	5875.331	5876.19	5875.657	5876.229	5879.033	5882.74	5884.494	5886.439	5890.917	5894.288
Niger	5603.004	5596.555	5591.527	5589.938	5591.79	5593.502	5597.563	5603.439	5611.245	5618.176
Pakistan	6643.466	6643.055	6641.989	6637.492	6630.551	6621.42	6611.33	6597.953	6583.643	6566.162
Papua New Guinea	7054.495	7046.92	7039.323	7030.035	7017.815	7003.001	6986.53	6965.967	6946.024	6924.594

Philippines	5916.699	5914.73	5911.17	5906.186	5898.974	5886.475	5875.453	5859.779	5844.611	5824.828
Sao Tome and Principe	6763.159	6753.086	6741.112	6729.74	6718.833	6709.439	6700.202	6692.4	6684.986	6673.37
Solomon Islands	6639.025	6641.333	6643.061	6646.294	6655.04	6663.818	6679.832	6695.94	6715.39	6736.505
Sri Lanka	5760.553	5771.039	5771.115	5763.848	5749.1	5727.525	5703.697	5672.67	5638.046	5600.323
Sudan	7338.313	7345.08	7346.474	7344.481	7342.958	7338.201	7331.324	7323.304	7313.006	7300.844
Timor-Leste	6049.537	6057.649	6060.315	6059.379	6058.393	6056.652	6053.177	6048.549	6043.528	6036.442
Tunisia	6575.643	6579.17	6580.264	6580.934	6580.97	6581.155	6581.492	6577.746	6576.629	6575.567
Ukraine	6211.035	6226.753	6238.663	6247.641	6257.453	6265.425	6276.668	6286.345	6294.842	6302.629
Uzbekistan	6739.024	6768.338	6784.525	6781.939	6764.527	6732.975	6686.433	6627.421	6555.718	6478.419
Vanuatu	7346.905	7333.654	7324.386	7322.157	7323.193	7325.934	7333.408	7341.492	7356.469	7368.23
Vietnam	5543.751	5531.408	5522.582	5514.87	5505.283	5493.75	5479.847	5466.008	5449.662	5432.397
Zambia	5848.281	5849.292	5850.528	5850.108	5848.852	5848.178	5846.619	5842.304	5838.704	5835.719

Age Standardized Prevalence rates- Low income countries (2008-2017)

Country Name	2008	2009	2010	2011	2012	2013	2014	2015	2016	2017
Afghanistan	7971.819	7986.715	7993.534	7995.353	8001.677	8009.141	8018.332	8026.576	8033.347	8037.174
Benin	5685.326	5679.883	5673.66	5668.507	5662.392	5656.615	5650.563	5644.911	5637.402	5630.425
Burkina Faso	5740.788	5723.941	5711.686	5705.089	5697.858	5692.638	5687.332	5684.545	5681.542	5681.424
Burundi	6020.237	6012.858	6011.11	6009.57	6008.198	6009.791	6009.206	6011.844	6016.153	6021.371
Central African Republic	5204.308	5211.629	5215.716	5220.006	5225.973	5231.488	5238.72	5247.747	5256.792	5263.58
Chad	6041.041	6051.223	6055.017	6057.404	6057.082	6053.545	6049.898	6044.44	6038.743	6029.445
Comoros	6096.298	6084.251	6069.746	6056.595	6045.376	6034.765	6022.198	6010.278	5999.588	5989.314
Democratic Republic of the Congo	5369.225	5363.875	5362.413	5363.229	5365.568	5368.258	5371.113	5377.262	5384.316	5390.711
Eritrea	5328.161	5322.833	5315.562	5310.981	5308.91	5305.188	5305.602	5306.255	5303.574	5303.149
Ethiopia	5495.013	5494.806	5493.745	5490.117	5485.472	5482.105	5478.661	5474.766	5470.426	5466.321
Guinea	5852.47	5857.394	5859.749	5861.022	5864.686	5866.021	5866.246	5865.576	5864.725	5863.43
Guinea-Bissau	5856.861	5850.453	5844.769	5837.066	5827.893	5819.673	5810.225	5799.225	5788.928	5777.6
Haiti	6472.424	6465	6459.202	6459.093	6462.624	6469.211	6481.549	6497.158	6515.181	6536.215
Liberia	6103.197	6104.936	6105.22	6103.261	6105.52	6107.917	6112.39	6115.364	6118.565	6122.163
Madagascar	6431.631	6417.335	6403.391	6388.954	6373.527	6358.029	6343.149	6328.653	6313.608	6297.986
Malawi	6274.885	6262.547	6252.154	6243.561	6234.469	6225.703	6219.303	6211.495	6204.206	6200.508
Mali	5653.83	5651.725	5647.648	5643.06	5640.666	5636.042	5628.931	5623.293	5619.453	5613.281
Mozambique	6329.728	6335.109	6338.588	6340.481	6342.96	6344.656	6347.033	6346.719	6347.58	6347.417
Nepal	5726.979	5725.936	5720.495	5715.628	5710.6	5704.361	5699.125	5693.421	5686.02	5679.757
Niger	5603.004	5596.555	5591.527	5589.938	5591.79	5593.502	5597.563	5603.439	5611.245	5618.176
North Korea	5759.96	5754.892	5752.748	5752.05	5753.649	5754.66	5755.244	5755.996	5756.162	5757.801
Rwanda	5309.251	5304.407	5301.936	5302.04	5302.578	5303.973	5306.783	5309.034	5314.605	5319.706
Senegal	6001.408	5998.731	5994.655	5991.958	5992.194	5995.643	6001.117	6010.086	6017.075	6027.853
Sierra Leone	6614.836	6615.395	6612.346	6605.924	6597.693	6584.453	6568.647	6550.445	6531.096	6509.989
South Sudan	5877.78	5875.268	5874.311	5870.698	5869.218	5867.424	5865.213	5865.28	5863.175	5864.589
Syria	7063.032	7066.963	7071.921	7072.358	7075.612	7080.123	7086.473	7093.644	7102.444	7108.29
Tajikistan	5906.722	5904.665	5902.649	5902.464	5903.548	5906.426	5913.282	5921.781	5931.65	5942.454
Tanzania	5511.741	5515.697	5520.01	5525.433	5531.983	5537.41	5544.659	5550.32	5557.561	5567.162
The Gambia	6037.084	6042.072	6042.799	6041.792	6039.556	6035.317	6029.916	6026.142	6019.292	6013.607
Togo	6007.513	5999.203	5992.422	5984.309	5975.763	5965.787	5954.671	5942.959	5931.475	5917.945
Uganda	5794.162	5779.951	5765.726	5753.47	5742.027	5728.937	5716.845	5704.957	5691.967	5679.597
Yemen	7317.481	7319.545	7318.387	7311.436	7303.854	7295.549	7285.218	7272.967	7257.932	7239.93

Zimbabwe	6228.634	6214.878	6203.735	6195.003	6178.548	6158.017	6136.331	6111.535	6084.999	6056.156
