# Supplementary material for: Disparities in Cardiovascular Research Output and Disease Outcomes among High-, Middle- and Low-Income Countries – An Analysis of Global Cardiovascular Publications over the Last Decade (2008–2017)
Source: Glob Heart. 2021 Jan 18;16(1):4. doi: 10.5334/gh.815 (PMC7845477; doi:10.5334/gh.815)
Supplement: Appendix D. — Age Standardized DALYs for countries in each income group (2008–2017). [file gh-16-1-815-s4.pdf]

Age Standardized DALYs- High Income countries (2008-2017)

Country Name	2008	2009	2010	2011	2012	2013	2014	2015	2016	2017
Andorra	2130.399	2110.288	2085.724	2059.535	2045.27	2033.464	2019.264	1997.875	1990.093	1980.266
Antigua and Barbuda	3987.829	3913.821	3878.583	3847.095	3807.688	3767.584	3772.175	3714.168	3690.619	3669.008
Argentina	4039.036	3985.67	3895.332	3842.03	3786.6	3720.58	3604.526	3564.017	3577.366	3561.935
Australia	2078.141	1999.92	1922.071	1866.971	1783.289	1742.629	1749.613	1751.732	1755.396	1783.149
Austria	2734.72	2744.94	2659.001	2581.419	2506.982	2442.858	2381.08	2349.421	2283.574	2268.608
Bahrain	3860.784	3597.936	3433.544	3329.557	3212.317	3091.364	3005.833	2985.487	2960.784	2948.826
Barbados	3379.567	3273.316	3225.728	3216.552	3173.22	3183.588	3213.713	3219.817	3216.833	3217.803
Belgium	2500.318	2421.237	2325.339	2284.227	2245.999	2189.58	2088.955	2080.808	2021.268	2000.58
Brunei	4409.068	4346.354	4268.048	4227.914	4185.387	4146.455	4104.763	4067.805	4045.118	4035.761
Canada	2245.075	2160.807	2071.859	2001.312	1995.102	1954.365	1993.993	2011.555	1985.403	1977.788
Chile	2613.99	2661.186	2639.301	2537.574	2486.163	2467.904	2397.551	2375.402	2367.115	2384.502
Croatia	5383.778	5039.911	4864.048	4692.348	4544.389	4273.519	4246.371	4332.199	4061.756	4010.986
Cyprus	3346.121	3172.409	2992.028	2830.577	2705.704	2634.453	2583.739	2542.652	2505.897	2497.136
Czech Republic	4760.321	4610.091	4463.997	4373.877	4261.861	4135.7	3945.833	3907.757	3824.329	3802.516
Denmark	2481.965	2394.93	2288.436	2142.727	2050.622	1981.893	1901.313	1859.592	1869.986	1883.958
Estonia	6288.946	5923.669	5404.793	5217.373	5066.743	4791.464	4689.405	4425.416	4361.108	4320.048
Finland	3212.731	3121.714	3033.874	2914.416	2825.213	2742.902	2629.459	2538.807	2545.572	2564.344
Germany	2886.989	2831.069	2749.075	2674.127	2606.539	2608.402	2513.912	2562.359	2597.752	2570.201
Greece	3530.114	3426.668	3311.722	3287.476	3259.634	3081.72	2976.009	2978.172	2991.893	3000.977
Hungary	6133.835	6061.243	5879.041	5708.331	5603.042	5373.042	5241.016	5438.22	5087.739	4949.813
Iceland	2213.465	2176.343	2089.476	2016.459	1993.717	1969.655	1953.652	1943.208	1915.531	1930.134
Ireland	2717.987	2666.946	2379.051	2364.395	2325.913	2249.253	2171.03	2129.641	2081.659	2074.523
Israel	1978.487	1860.013	1787.25	1775.655	1717.95	1684.911	1637.58	1636.652	1597.255	1583.888
Italy	2188.527	2140.512	2050.637	2026.67	1988.734	1924.404	1865.039	1882.206	1799.402	1763.827
Japan	1906.018	1865.696	1842.153	1826.401	1768.099	1724.858	1681.351	1629.318	1621.795	1619.132
Kuwait	4320.47	4029.702	3663.205	3416.754	3340.497	3224.856	3096.804	3062.098	3052.013	3047.557
Latvia	8349.646	7880.479	7735.085	7300.668	7075.942	7032.34	6781.828	6574.697	6543.053	6475.453
Lithuania	7650.342	6883.059	6796.702	6637.49	6492.432	6468.548	6159.147	6158.192	6096.463	6028.354
Luxembourg	2735.673	2637.355	2557.694	2477.122	2405.681	2321.362	2265.764	2224.198	2188.668	2171.061
Malta	3129.658	3005.286	2920.738	2841.7	2793.671	2688.842	2635.301	2631.583	2694.622	2719.043
Netherlands	2249.042	2134.877	2053.233	1990.308	1943.666	1877.686	1805.887	1801.215	1797.742	1804.227
New Zealand	2575.803	2476.327	2376.344	2328.508	2272.795	2206.271	2190.2	2158.076	2167.227	2175.198

Norway	2352.972	2286.575	2217.031	2198.347	2057.798	2032.783	1924.55	1845.143	1834.627	1846.367
Oman	6661.195	6465.668	6482.093	6516.378	6302.938	5994.97	5852.419	5739.889	5468.025	5411.323
Panama	2789.276	2797.869	2740.4	2724.303	2707.827	2673.634	2623.951	2552.69	2507.685	2479.141
Poland	5253.327	5096.979	4834.138	4684.64	4602.415	4433.442	4216.318	4215.277	4135.02	4072.308
Portugal	2626.757	2518.994	2406.519	2300.127	2225.542	2159.668	2109.237	2033.098	2050.434	2071.535
Qatar	4205.106	3984.348	3769.604	3607.811	3471.489	3348.407	3236.74	3139.224	3072.611	3054.086
Saudi Arabia	5886.563	5941.325	5954.681	5892.624	5764.98	5605.35	5417.58	5244.715	5095.057	4967.518
Seychelles	5463.882	5404.456	5307.417	5227.719	5174.637	5080.393	5029.758	5017.729	4975.179	4945.03
Singapore	2747.392	2545.459	2445.412	2383.643	2297.538	2208.327	2128.868	2060.582	1990.124	1966.682
Slovakia	6382.382	6164.226	5859.091	5656.617	5416.871	5211.57	5067.507	5119.744	4916.35	4836.709
Slovenia	3177.315	3043.589	2914.396	2793.919	2761.285	2680.114	2514.409	2546.068	2506.117	2501.771
Spain	2205.889	2092.165	2002.818	1955.279	1907.273	1838.694	1806.606	1824.561	1769.339	1745.554
Sweden	2711.602	2639.793	2517.223	2469.848	2387.923	2331.377	2265.783	2210.644	2159.107	2151.729
Switzerland	1897.005	1871.751	1807.644	1731.515	1706.684	1674.121	1608.608	1570.152	1523.228	1504.542
The Bahamas	4965.329	5019.055	5007.42	4996.716	4908.869	4872.189	4884.796	4907.17	4896.873	4882.408
Trinidad and Tobago	5286.085	5000.526	4753.599	4497.966	4463.304	4556.051	4578.549	4591.342	4677.145	4707.153
United Arab Emirates	6071.009	6048.697	6058.102	6089.139	6113.196	6117.078	6123.188	6116.75	6109.181	6084.02
United Kingdom	2685.33	2544.484	2443.233	2323.348	2246.507	2231.952	2181.651	2191.079	2193.028	2201.994
United States	3262.066	3177.392	3068.427	3049.103	3007.282	2984.585	2974.896	2979.461	3017.412	3029.691
Uruguay	3469.016	3311.896	3240.613	3235.428	3177.679	3084.274	2975.69	2972.3	2973.863	2988.339

Age Standardized DALYs- Upper middle Income countries (2008-2017)

Country Name	2008	2009	2010	2011	2012	2013	2014	2015	2016	2017
Albania	5753.062	5610.413	5498.938	5456.842	5404.499	5377.99	5373.124	5325.109	5272.025	5227.748
Algeria	5720.886	5601.219	5499.42	5418.446	5314.406	5234.597	5162.555	5081.103	4969.993	4888.275
Armenia	6973.181	6841.302	6667.913	6436.226	6206.424	5961.478	5837.123	5782.492	5732.665	5735.425
Azerbaijan	10956.55	10779.9	10674.64	10534.65	10354.9	10097.09	9912.667	9759.502	9663.565	9660.424
Belarus	10186.36	10184.89	10217.34	10286.67	9056.067	8846.563	8543.018	8071.676	7909.417	7892.492
Belize	3986.659	3948.68	3881.256	3813.065	3743.185	3707.134	3708.756	3706.974	3716.79	3709.499
Bosnia and Herzegovina	5868.306	5859.465	5768.033	5697.285	5576.306	5560.083	5512.787	5560.066	5482.549	5411.824
Botswana	4604.909	4685.213	4659.001	4649.301	4548.459	4485.878	4434.514	4350.453	4200.518	4115.869
Brazil	4283.002	4204.05	4127.806	4051.132	3939.572	3849.396	3748.297	3691.797	3704.752	3735.02
Bulgaria	8835.409	8603.299	8428.563	8235.735	7930.835	7736.274	7955.539	7785.208	7741.358	7732.12
China	4709.143	4758.259	4803.317	4803.142	4777.972	4692.8	4722.783	4763.75	4717.811	4575.859
Colombia	3002.261	2956.537	2833.176	2651.604	2575.543	2500.543	2452.984	2445.295	2435.064	2423.235
Costa Rica	2479.665	2465.021	2661.428	2564.566	2528.002	2494.883	2485.255	2554.525	2677.057	2690.021
Cuba	3758.751	3726.087	3697.309	3450.626	3498.328	3490.313	3494.168	3574.518	3577.064	3541.611
Dominica	4130.995	4129.635	4157.694	4238.71	4327.786	4392.918	4460.031	4441.808	4426.588	4406.661
Dominican Republic	4712.864	4713.667	4753.083	4727.503	4881.273	5101.251	5307.957	5455.48	5492.8	5396.689
Ecuador	3239.478	3143.079	3084.657	3022.965	2942.41	2825.852	2750.712	2706.408	2719.747	2704.243
Equatorial Guinea	4418.543	4305.032	4166.206	4027.663	3945.892	3850.304	3801.356	3767.947	3745.371	3710.822
Fiji	10007.08	9774.289	9681.291	9488.462	9421.496	9366.156	9301.492	9233.845	9169.386	9097.926
Gabon	6037.297	5871.015	5647.642	5551.877	5427.794	5288.179	5195.936	5115.702	5021.325	4948.71
Grenada	4804.589	4832.858	4864.144	4885.173	4845.926	4852.382	4889.972	4873.966	4911.43	4866.6
Guatemala	2885.133	2989.267	2965.003	2934.844	2900.684	2850.679	2884.629	2881.073	2958.942	2965.716
Guyana	7865.022	7714.114	8113.271	8462.956	8605.606	8324.574	8173.223	8004.061	7856.515	7726.969
Iran	5415.476	5314.648	5200.651	5099.357	4999.067	4913.928	4850.645	4822.5	4749.235	4670.861
Iraq	7884.013	7360.704	6907.217	6377.966	5908.125	5592.463	5219.691	4840.185	4647.09	4551.413
Jamaica	3512.865	3744.961	3520.188	3502.573	3628.758	3879.93	3989.806	3988.769	4090.156	4048.506
Jordan	4712.922	4479.956	4292.959	4154.061	4023.98	3913.43	3875.895	3809.278	3777.858	3759.508
Kazakhstan	12049.03	11217.96	11083.84	10734.86	10304.47	9727.505	9205.152	8888.778	8794.576	8627.844
Libya	6310.009	6669.129	6736.598	6737.538	6622.49	6675.034	6764.039	6783.888	6848.586	6881.59
Macedonia	6936.727	6787.887	6600.221	6327.329	6195.061	5867.93	5714.389	5916.807	5918.01	5880.094
Malaysia	5664.765	5734.751	5610.779	5343.669	5262.75	5181.478	5304.718	5349.305	5352.684	5370.058
Maldives	4563.111	4168.695	3934.661	3686.511	3479.947	3365.124	3271.073	3220.613	3181.897	3162.238

Marshall Islands	14453.15	14276.54	14145.55	13910.73	13700.95	13508.92	13298.89	13138.24	12924.35	12757.65
Mauritius	5371.539	5165.332	4859.477	4646.238	4587.71	4434.923	4456.244	4495.844	4493.297	4465.121
Mexico	2876.133	2934.801	2903.698	2840.358	2825.664	2825.669	2840.486	2829.368	2865.455	2862.04
Montenegro	7110.409	6874.417	6680.326	6601.576	6542.557	6536.91	6485.548	6472.871	6451.227	6424.79
Namibia	5858.641	5490.24	5179.681	4892.138	4737.89	4622.058	4533.481	4470.925	4421.555	4386.435
Paraguay	4208.838	4323.181	4332.098	4210.121	4105.401	4131.009	3977.642	3903.705	3872.327	3832.539
Peru	2250.553	2341.2	2312.247	2214.311	2112.86	2014.825	1906.419	1837.783	1820.007	1812.458
Romania	7674.986	7581.38	7369.999	6831.268	6723.526	6495.452	6452.845	6344.278	6282.816	6264.816
Russian Federation	11979.97	11183.74	11034.31	10024.05	9498.043	9103.376	9026.216	8696.464	8391.231	8258.936
Saint Lucia	4046.382	3993.601	3960.735	3901.223	3908.534	3934.329	3919.988	3959.89	3978.2	4004.957
Saint Vincent and the Grenadines	4946.954	4916.709	4901.741	4947.752	5030.7	5100.903	5158.615	5141.436	5117.24	5102.27
Samoa	6960.679	7030.472	7012.536	7008.415	7000.864	6977.336	6956.379	6932.538	6903.91	6876.691
Serbia	7642.834	7523.755	7240.005	7154.554	7055.997	6795.843	6741.562	6701.989	6428.699	6300.519
South Africa	5577.484	5364.227	5083.511	4783.358	4543.98	4333.297	4204.529	4146.681	4052.238	3797.177
Suriname	5726.955	5532.495	5456.433	5320.03	5211.86	5204.71	5248.193	5375.924	5379.247	5335.204
Thailand	2948.909	2810.971	2778.168	2708.734	2636.44	2561.775	2545.115	2536.267	2576.613	2572.88
Tonga	5124.853	5109.253	5111.812	5061.167	5019.967	4984.89	4945.115	4896.885	4842.426	4805.223
Turkey	3813.935	3879.208	3826.599	3750.284	3685.832	3675.065	3672.196	3585.569	3482.318	3418.384
Turkmenistan	13180.32	11763.76	10978.73	10790.36	10800.61	10961.41	11066.06	11001.31	10909.02	10860.51
Venezuela	4068.604	3993.301	3841.27	3816.099	3833.357	3809.798	3902.446	3927.258	3837.764	3870.854

Age Standardized DALYs- Lower middle Income countries (2008-2017)

Country Name	2008	2009	2010	2011	2012	2013	2014	2015	2016	2017
Angola	6662.419	6490.774	6311.616	6158.78	6027.64	5876.699	5619.337	5516.344	5421.447	5358.069
Bangladesh	7480.382	7376.21	7353.83	7020.445	6720.641	6469.93	6292.825	6115.04	6029.094	5975.182
Bhutan	4802.382	4757.419	4692.475	4628.491	4566.317	4513.411	4458.677	4402.328	4343.761	4284.978
Bolivia	4285.416	4212.278	4142.308	4073.705	4007.756	3947.596	3893.821	3846.433	3802.748	3766.15
Cambodia	6065.874	5922.995	5799.673	5692.555	5591.251	5501.155	5433.22	5375.407	5320.876	5270.173
Cameroon	5940.115	5855.618	5761.135	5642.053	5514.783	5376.112	5239.351	5115.854	5000.226	4875.596
Cape Verde	3840.132	3804.021	3776.353	3742.721	3729.464	3685.422	3645.664	3606.013	3598.663	3568.077
Congo	7774.03	7591.281	7441.736	7188.469	7072.726	6942.687	6867.775	6784.091	6658.647	6623.179
Cote d'Ivoire	7284.705	7119.112	6999.709	6895.54	6836.254	6757.58	6661.18	6530.585	6365.373	6219.408
Djibouti	5730.543	5657.767	5573.679	5480.676	5404.403	5329.922	5285.514	5218.367	5166.409	5105.927
Egypt	10495.04	10697.52	10738.07	10581.01	10586.68	10245.97	10120.8	10258.95	10188	10148.52
El Salvador	3399.189	3460.234	3339.736	3332.157	3159.043	3194.626	3337.146	3335.923	3301.619	3265.481
Federated States of Micronesia	10303.37	10289.91	10264.48	10205.89	10148.11	10103.76	10079.29	10048.84	9993.907	9941.915
Georgia	9168.606	9385.116	9247.596	8932.29	8542.814	8341.717	8120.411	8104.048	8416.212	8505.723
Ghana	6589.874	6531.065	6461.259	6394.345	6286.827	6180.333	6080.173	6004.328	5921.41	5808.053
Honduras	5004.183	4934.68	4880.679	4861.258	4815.132	4789.991	4778.531	4733.724	4686.761	4641.963
India	5731.126	5781.185	5863.937	5933.427	5957.751	5918.293	5882.653	5862.28	5882.632	5804.264
Indonesia	7171.855	7171.44	7144.291	7099.857	7076.092	7043.678	7009.263	6978.65	6873.967	6777.336
Kenya	4983.126	4919.401	4847.443	4770.924	4674.282	4559.728	4460.748	4387.248	4324.329	4256.49
Kiribati	11567.41	11555.54	11536.8	11472.18	11388.82	11292.62	11192.82	11075.81	10949.2	10832.44
Kyrgyzstan	10503.77	10019.89	9782.41	9625.971	9429.285	9174.758	8947.785	8583.496	8191.501	8066.389
Laos	9664.836	9394.653	9103.533	8784.89	8561.6	8350.153	8147.03	7951.72	7776.255	7627.015
Lesotho	9017.69	8893.794	8871.664	8751.768	8746.728	8741.905	8650.068	8522.97	8193.905	7846.216
Mauritania	4949.845	4884.599	4803.674	4747.512	4694.686	4639.856	4577.007	4523.267	4473.616	4425.877
Moldova	9425.155	9453.049	9406.811	8577.782	8207.845	7884.803	8055.445	8124.619	7699.875	7449.824
Mongolia	11680.85	11701.86	11283.68	10779.5	10208.11	9709.661	9289.28	9059.956	8957.314	8890.933
Morocco	9051.881	8893.551	8747.807	8609.706	8425.167	8287.553	8163.822	8032.177	7909.77	7789.301
Myanmar	5280.957	5089.989	4891.287	4732.991	4583.25	4464.276	4344.385	4255.935	4169.941	4091.919
Nicaragua	3033.829	2939.616	2802.215	2808.811	2807.482	2806.579	2873.085	2722.318	2632.734	2579.629
Niger	4826.901	4776.495	4709.916	4701.8	4690.298	4699.714	4722.56	4739.862	4757.519	4733.315
Pakistan	8932.416	8825.877	8729.293	8648.107	8577.43	8513.175	8469.174	8390.897	8319.647	8222.879
Papua New Guinea	16047.13	15924.99	15793.47	15629.83	15452.94	15267.3	15051.99	14835.48	14659.32	14493.62

Philippines	7449.183	7580.464	7584.474	7606.781	7644.872	7648.665	7618.407	7719.98	7612.523	7493.429
Sao Tome and Principe	5506.037	5502.786	5492.803	5488.532	5574.394	5534.242	5471.526	5438.926	5422.72	5375.207
Solomon Islands	10985.37	10915.27	10828.48	10694.67	10571.58	10464.48	10369.18	10284.19	10194.82	10123.65
Sri Lanka	4798.851	4526.354	4418.253	4372.36	4245.712	4067.35	4002.59	3899.711	3797.999	3717.433
Sudan	9950.064	9782.927	9610.652	9416.314	9262.597	9103.135	8959.121	8816.897	8682.817	8576.398
Timor-Leste	6491.355	6408.156	6346.614	6318.743	6308.694	6350.159	6427.342	6461.079	6487.057	6517.483
Tunisia	5694.133	5593.91	5498.619	5430.195	5366.334	5310.175	5255.989	5201.011	5144.237	5084.058
Ukraine	11786.37	10500.79	10042.98	9594.843	9590.148	9572.213	9171.155	10973.77	10658.16	10051.21
Uzbekistan	13633.6	13337.19	13202.7	13044.09	13011.73	12925.15	12693.36	12525.19	12305.78	12113.28
Vanuatu	12853.47	12754.57	12630.49	12537.34	12466.1	12396.33	12346.29	12321.57	12295.22	12254.98
Vietnam	5191.637	5134.456	5070.91	4990.477	4918.804	4860.762	4812.364	4764.564	4719.105	4680.629
Zambia	5374.14	5254.81	5224.666	5134.091	5027.53	4927.976	4837.511	4772.419	4711.203	4674.697

Age Standardized DALYs- Low Income countries (2008-2017)

Country Name	2008	2009	2010	2011	2012	2013	2014	2015	2016	2017
Afghanistan	15381.7	14996.43	14642.84	14326.49	14005.41	13720.11	13471.85	13271.98	13091.25	12951.29
Benin	5024.58	4998.144	4966.436	4969.713	4968.346	4923.185	4853.303	4777.224	4697.321	4623.297
Burkina Faso	5491.391	5514.956	5485.395	5467.232	5495.185	5486.957	5466.932	5431.879	5389.224	5321.61
Burundi	6451.039	6280.197	6185.519	6122.354	6053.181	5966.755	5886.785	5815.291	5737.237	5701.59
Central African Republic	9570.542	9552.41	9571.291	9523.61	9413.317	9457.349	9439.066	9419.78	9353.534	9236.369
Chad	5934.058	5950.008	5983.712	5956.091	5913.069	5834.983	5782.785	5735.892	5698.693	5644.731
Comoros	5583.802	5495.762	5412.838	5374.406	5321.805	5264.643	5216.083	5166.006	5118.967	5077.726
Democratic Republic of the Congo	6663.652	6639.013	6604.553	6584.715	6548.635	6452.907	6363.729	6280.405	6197.494	6140.639
Eritrea	7364.376	7246.363	7187.995	7033.92	6877.284	6729.476	6597.63	6546.661	6389.502	6236.42
Ethiopia	4671.227	4471.154	4263.676	4111.369	3976.321	3830.814	3740.144	3668.06	3599.619	3549.551
Guinea	6793.878	6872.907	6957.365	6976.613	6993.669	7009.2	7040.83	6989.356	6902.529	6804.023
Guinea-Bissau	9134.367	9056.572	8924.683	8761.869	8652.779	8552.013	8440.169	8324.251	8191.165	8039.486
Haiti	9468.791	9357.182	9353.413	9227.289	9122.922	9017.351	8921.892	8828.245	8745.764	8661.772
Liberia	5695.525	5740.53	5756.682	5719.594	5744.796	5703.63	5607.793	5541.254	5487.103	5436.896
Madagascar	9105.692	9123.27	9105.27	9050.342	8961.624	8864.983	8779.045	8699.387	8595.192	8499.178
Malawi	5317.011	5190.854	5128.889	5014.259	4904.576	4803.202	4716.784	4659.414	4607.519	4540.56
Mali	5469.699	5415.289	5374.805	5307.77	5259.808	5227.391	5204.305	5196.587	5212.656	5159.63
Mozambique	7573.189	7565.548	7580.505	7529.829	7466.598	7364.45	7259.237	7148.835	7014.18	6861.497
Nepal	5173.667	5219.154	5248.551	5306.639	5331.655	5330.505	5303.38	5311.155	5283.112	5242.176
Niger	4826.901	4776.495	4709.916	4701.8	4690.298	4699.714	4722.56	4739.862	4757.519	4733.315
North Korea	6750.933	6726.257	6707.515	6688.113	6669.022	6654.285	6625.346	6597.911	6552.283	6491.096
Rwanda	4097.178	3920.904	3809.117	3722.8	3665.193	3631.068	3598.779	3559.532	3519.703	3490.104
Senegal	4893.738	4845.502	4839.827	4843.184	4840.882	4843.164	4843.835	4835.096	4797.673	4758.279
Sierra Leone	7557.632	7532.772	7527.133	7419.579	7339.009	7168.505	7030.213	6919.748	6802.995	6704.524
South Sudan	6130.482	6036.274	5949.687	5869.81	5821.466	5788.447	5748.125	5746.622	5722.691	5686.723
Syria	7903.835	7709.337	7562.123	7485.645	7460.148	7480.014	7497.247	7490.326	7481.743	7437.471
Tajikistan	8449.25	8401.665	8360.271	8346.59	8236.303	8127.996	8147.64	8235.335	8294.319	8270.232
Tanzania	4422.06	4373.966	4328.557	4284.072	4299.604	4310.888	4291.353	4280.905	4259.757	4240.93
The Gambia	6825.685	6803.147	6774.593	6766.742	6765.613	6752.211	6722.739	6680.38	6618.232	6559.815
Togo	6282.076	6229.657	6201.219	6129.701	6055.759	5953.339	5850.451	5744.253	5650.288	5574.39
Uganda	5003.94	4818.842	4645.621	4511.754	4427.951	4379.729	4313.291	4201.656	4108.8	4063.379
Yemen	10631.98	10437.81	10225.85	10090.2	9966.759	9864.806	9792.959	9807.498	9833.051	9855.103

Zimbabwe	8326.922	8288.341	8067.467	7674.59	7313.221	7008.73	6764.138	6548.697	6375.354	6238.641
