# Supplementary material for: Disparities in Cardiovascular Research Output and Disease Outcomes among High-, Middle- and Low-Income Countries – An Analysis of Global Cardiovascular Publications over the Last Decade (2008–2017)
Source: Glob Heart. 2021 Jan 18;16(1):4. doi: 10.5334/gh.815 (PMC7845477; doi:10.5334/gh.815)
Supplement: Appendix E. — Age Standardized Death rates for countries in each income group (2008–2017). [file gh-16-1-815-s5.pdf]

Age Standardized Death rates- High Income countries (2008-2017)

Country Name	2008	2009	2010	2011	2012	2013	2014	2015	2016	2017
Andorra	115.2623	114.6733	113.7327	112.7481	112.2441	112.0412	111.4779	110.4938	109.8791	109.1345
Antigua and Barbuda	211.7635	207.5422	205.5267	203.3598	200.665	198.2481	197.8317	194.3107	192.8148	191.5109
Argentina	208.6275	206.3622	204.9061	203.1583	200.3557	197.9608	193.1411	192.1973	193.1299	191.0321
Australia	128.8712	123.1132	117.453	114.2274	108.5537	105.0895	105.1701	105.3454	105.9814	107.7914
Austria	176.0832	174.683	167.3824	163.1627	159.0439	154.8346	151.0529	149.3015	145.4492	145.1827
Bahrain	208.4914	192.4356	182.6156	176.2335	168.7495	161.3976	156.2139	154.674	152.651	151.689
Barbados	174.6198	168.1276	167.1036	167.6509	165.6818	166.7464	168.8772	169.6872	169.704	170.0497
Belgium	144.9872	140.4508	134.3033	131.4917	129.5659	126.5405	119.7165	119.8508	116.1126	114.8976
Brunei	219.1366	216.2188	212.2083	210.3747	208.3361	206.3624	204.1297	202.3457	201.4846	201.2846
Canada	124.4497	119.0095	113.3616	109.0683	108.1467	104.8801	106.4622	107.3028	105.9532	105.5993
Chile	141.1612	143.502	143.555	137.5582	134.5105	132.7628	128.285	127.0566	126.6239	127.9929
Croatia	338.3454	317.5806	308.9121	297.8889	287.9292	271.7611	270.2572	275.8514	257.1542	253.7821
Cyprus	194.4085	183.1494	171.4183	161.9521	154.1645	149.35	146.594	144.1677	142.0852	141.1708
Czech Republic	289.4651	279.2687	268.1608	261.2769	255.0737	247.7253	236.2921	234.935	229.2269	227.4849
Denmark	150.3219	145.3191	138.5619	129.2064	123.7471	119.681	114.5941	112.452	113.6298	114.7673
Estonia	360.1613	343.5089	316.7474	305.6447	296.1915	283.1585	275.2916	261.3363	258.3996	255.5693
Finland	187.2509	182.7411	177.8675	170.8164	166.323	161.4916	155.3772	150.9171	151.9963	153.5066
Germany	173.196	169.8537	164.8143	160.8446	157.5967	158.753	153.0305	156.7182	159.4687	156.1385
Greece	217.2155	206.8261	199.2264	196.8634	195.1276	182.7938	174.4825	173.3689	174.8579	175.6948
Hungary	339.9652	338.9602	332.4615	324.3801	319.158	306.5562	296.4801	305.3037	285.4094	278.2961
Iceland	137.5107	135.206	129.5061	125.3802	123.7781	122.4272	121.2159	119.965	117.997	117.9921
Ireland	163.9279	159.6953	142.28	142.0708	141.293	137.4663	132.3391	130.1327	127.0777	126.4586
Israel	119.6226	111.8483	106.8152	106.5725	102.4829	99.87729	97.09788	96.72663	94.0857	93.31981
Italy	138.9116	136.2877	130.5663	129.1328	126.803	122.0703	118.3221	120.6429	115.4596	113.151
Japan	93.80241	91.1717	90.11394	89.78451	86.83023	84.51895	82.15665	79.37475	79.35764	79.37036
Kuwait	189.3777	175.6471	159.1733	147.7928	144.1777	138.4774	132.4024	131.5929	131.7587	132.2351
Latvia	433.7702	413.4436	406.9801	388.516	380.7538	380.826	367.3873	358.3737	356.6328	350.0599
Lithuania	420.469	385.0979	383.4679	375.651	368.4408	367.903	352.1904	353.2234	348.2004	342.9892
Luxembourg	160.2151	155.2855	151.1845	146.3989	142.406	137.4531	134.1649	131.6103	129.0628	128.2751
Malta	197.2393	190.0209	183.9581	178.5474	175.5082	167.9384	163.8382	163.4849	167.4933	168.7107
Netherlands	133.8315	127.0416	122.2636	118.8889	116.5724	112.8316	108.6633	108.9649	109.2002	109.3614
New Zealand	155.2432	148.8295	142.5854	140.74	137.0801	132.0288	131.5909	129.6507	128.9521	128.7973

Norway	146.2876	141.4425	137.3636	137.3322	127.7506	125.7909	119.0826	114.0514	113.5466	114.3157
Oman	320.9576	314.8811	320.98	327.7619	317.0446	299.4423	292.3834	285.7272	268.6023	266.3415
Panama	139.8191	139.9748	138.244	135.4047	134.1566	133.7399	131.0449	128.5239	128.6743	128.3461
Poland	293.6871	284.983	268.1034	258.748	254.8874	246.7756	235.8081	236.1324	231.3031	227.3309
Portugal	162.3464	155.4066	147.9371	140.1919	136.0452	131.4137	127.7667	124.576	126.3559	127.8422
Qatar	247.3829	233.6336	220.8197	211.0384	202.6178	194.7543	187.6014	181.9566	177.8391	176.6898
Saudi Arabia	305.6672	309.007	310.6985	309.217	303.6555	295.2308	284.2429	274.6964	266.6718	259.538
Seychelles	270.6019	270.2075	265.8648	261.9364	258.7136	252.4876	248.4676	247.4	244.7863	242.6481
Singapore	134.9887	122.8803	116.7366	113.1009	109.099	103.789	99.83472	96.31142	92.58692	92.24346
Slovakia	370.3929	360.8356	344.5842	333.2294	320.0938	308.5912	299.2575	303.2657	292.2996	287.959
Slovenia	194.9794	188.6106	180.8711	173.2762	172.1414	167.9308	156.971	157.2871	154.1939	153.4934
Spain	127.0985	120.0597	114.3888	111.9279	109.2744	104.7686	102.7833	104.7323	101.2341	99.40349
Sweden	169.2202	164.154	156.1527	153.604	148.7403	144.6716	140.2105	136.7982	134.0919	133.982
Switzerland	127.2924	125.29	120.7284	114.8138	113.0262	110.372	105.7838	104.2296	101.6597	99.73932
The Bahamas	239.9959	243.7502	242.4645	242.4397	237.0113	235.0366	235.0962	236.9815	236.5669	235.9542
Trinidad and Tobago	257.7069	244.4143	231.4734	214.7092	211.7099	219.2432	220.6053	220.8334	227.0618	228.467
United Arab Emirates	325.3183	322.7223	322.2391	321.9385	322.1766	321.5072	321.1363	320.2391	319.3754	317.8397
United Kingdom	151.146	142.0408	135.9897	129.2596	126.1348	125.2388	121.6865	122.3063	121.6966	122.1372
United States	167.1522	161.9411	156.1114	154.9615	152.3738	150.7277	149.6784	149.3207	150.5341	151.0895
Uruguay	185.4389	175.4086	171.8371	175.3387	172.1632	165.8276	159.3341	159.5283	159.9259	160.7084

Age Standardized Death rates- Upper Middle Income countries (2008-2017)

Country name	2008	2009	2010	2011	2012	2013	2014	2015	2016	2017
Albania	336.9151	328.2437	322.9101	319.5235	316.3277	314.5838	313.6391	310.4933	307.1128	304.1954
Algeria	321.7407	316.0801	312.1666	308.2453	302.6015	298.027	293.7284	289.0246	282.5895	278.3639
Armenia	404.1899	396.2588	387.932	376.0226	364.1051	351.8897	345.0186	341.535	339.4903	341.0098
Azerbaijan	622.4219	611.7261	609.1467	605.5032	594.413	577.0034	569.2291	561.3328	556.9871	559.8121
Belarus	529.8612	532.6357	532.9338	536.5877	485.8399	481.2073	467.4327	447.6967	443.9504	443.1292
Belize	196.5093	194.2569	188.9695	184.5123	179.8021	178.1179	178.3041	177.8275	177.0288	176.9573
Bosnia and Herzegovina	337.2964	344.7582	342.1082	339.5564	334.6852	334.4965	334.066	338.9084	333.7344	329.6346
Botswana	253.4542	260.2092	261.2581	262.3706	258.3528	256.1545	254.2256	250.0778	241.8905	237.372
Brazil	201.9913	198.4519	195.5421	192.2608	186.6537	182.4662	178.0148	175.9413	176.6137	177.9606
Bulgaria	492.8907	477.9616	471.6961	461.4118	445.3816	431.9586	442.944	432.0115	426.0957	424.6881
China	273.8712	279.4101	283.8327	284.0114	280.5499	271.8287	271.1663	271.1419	268.0772	261.8991
Colombia	156.9041	153.2299	146.5683	136.7838	132.4049	128.4023	125.6792	125.6327	125.0977	124.2398
Costa Rica	124.5825	123.1618	132.9113	128.3784	127.0398	125.5241	125.6537	129.9302	136.8902	137.9732
Cuba	203.5257	201.9916	200.6366	186.2974	187.4821	186.9935	188.2587	193.1026	193.2357	190.9682
Dominica	214.1783	212.8968	213.8843	217.628	222.4317	225.8035	230.0868	229.7572	228.674	227.376
Dominican Republic	231.7355	232.1918	234.325	232.5872	240.337	251.5841	262.2084	269.4831	271.4306	266.653
Ecuador	166.1898	159.6948	157.064	155.3875	152.1425	146.4864	143.4002	141.4747	141.8467	140.4478
Equatorial Guinea	240.5267	234.2717	227.427	221.1333	216.8067	211.8543	208.103	206.5315	205.1368	202.8121
Fiji	454.2384	445.1527	441.5645	432.6727	428.972	425.5689	422.0868	418.3935	415.3743	412.8204
Gabon	310.5197	302.5784	292.6554	288.2792	282.3129	275.6288	271.1243	267.3336	263.2048	259.9667
Grenada	244.672	246.8319	248.869	249.5312	245.3488	245.7309	247.258	245.5944	246.9333	243.9639
Guatemala	154.5075	160.1605	158.9956	157.795	156.0654	152.8257	154.0593	153.0415	156.2472	155.8977
Guyana	375.7256	368.4665	386.8467	403.6667	410.3259	397.3869	391.3552	384.6005	378.6517	373.1588
Iran	295.0621	290.0657	285.8827	281.1586	275.3436	271.6486	271.4011	273.1128	272.3687	270.3077
Iraq	384.813	359.1619	338.5322	311.4318	288.1153	275.2609	254.7061	232.9021	222.72	218.6121
Jamaica	181.4244	193.3219	182.6421	179.1565	183.4702	197.118	202.5363	201.438	208.6857	206.5373
Jordan	248.837	238.8942	232.8698	226.9202	220.6527	215.5778	214.2224	210.3594	208.8669	208.2568
Kazakhstan	605.0006	570.5642	568.0561	558.013	542.0063	517.5382	495.5192	482.1751	478.2346	466.7918
Libya	304.598	333.7239	342.374	336.3601	329.5508	334.5099	338.7911	336.7364	339.9319	341.8622
Macedonia	400.0408	387.8884	375.5576	357.9909	348.0273	326.2761	313.7159	322.7601	322.5582	322.6881
Malaysia	286.4937	288.8433	280.7066	262.2114	256.2148	250.3223	255.474	257.7271	258.7151	260.9423
Maldives	248.7158	223.8313	210.7665	196.1964	183.8149	177.5326	171.8516	168.4307	166.1878	164.9051

Marshall Islands	627.2965	618.0004	615.1401	603.4077	594.8088	589.1622	578.611	575.1416	564.4997	557.7926
Mauritius	262.9032	249.5756	234.7378	224.5265	226.5077	221.0817	222.4004	223.4589	225.6893	224.6442
Mexico	149.4874	152.6259	152.0331	148.8895	148.866	150.8245	152.5696	152.5523	154.1592	152.7829
Montenegro	407.1052	389.0179	379.069	379.9945	383.5034	386.1844	384.799	387.2397	388.2318	387.3046
Namibia	317.6757	299.3764	283.4463	268.9714	261.7635	255.8258	251.5661	248.4844	245.9552	243.8106
Paraguay	208.8114	215.9962	220.1365	215.7631	211.3518	215.5176	207.4711	203.4607	201.2873	199.1279
Peru	105.6291	109.5677	108.6502	104.0651	100.0185	95.65385	90.02652	86.48304	85.52351	85.75458
Romania	452.835	448.9594	438.659	410.6244	404.1437	388.9679	385.332	379.5767	373.4033	370.9463
Russian Federation	601.3386	566.1767	559.2963	513.1887	489.6382	471.0364	465.9564	452.4579	439.0592	431.2975
Saint Lucia	207.0581	204.0725	201.9421	197.8071	198.4097	199.7986	198.1753	201.0346	202.5637	204.6199
Saint Vincent and the Grenadines	251.7599	249.2193	247.6327	248.8841	252.6464	256.0434	258.3194	257.0919	254.489	252.6754
Samoa	343.3471	351.4523	352.0949	354.3825	354.6501	353.8017	353.047	352.0222	350.6077	348.9773
Serbia	488.8335	487.7499	475.3697	477.8116	479.329	465.2372	463.8286	461.9567	446.5277	439.415
South Africa	285.1441	278.2547	266.4758	254.1185	243.7709	233.4267	226.5848	223.9414	217.9613	200.3805
Suriname	268.023	257.9376	253.9581	248.0923	243.8072	245.4691	251.92	262.9356	264.7084	258.3135
Thailand	133.4393	126.1846	124.345	120.0098	114.921	110.5204	109.0297	107.9601	109.4834	109.8612
Tonga	245.708	244.265	244.1543	241.4929	239.2442	237.389	235.2561	232.6599	229.7345	227.4903
Turkey	183.6404	188.1805	186.9624	184.8499	182.8745	183.6648	184.5383	180.4059	174.7572	171.2854
Turkmenistan	668.5819	600.1762	560.8839	550.7172	548.8051	551.1693	551.6723	545.9535	539.3308	536.7835
Venezuela	204.0806	200.679	195.0271	194.8944	195.8859	199.2874	212.8216	216.8741	203.7885	204.8503

Age Standardized Death rates- Lower Middle Income countries (2008-2017)

Country name	2008	2009	2010	2011	2012	2013	2014	2015	2016	2017
Angola	336.4515	328.7227	320.4133	313.3853	307.106	300.0315	287.9931	283.1517	279.1456	276.0446
Bangladesh	379.7372	376.6877	376.8675	356.1783	336.4253	322.8198	313.3434	303.1673	299.2407	298.0025
Bhutan	239.07	237.5101	235.0588	232.6285	230.11	227.8164	225.3159	222.7243	219.9492	217.0658
Bolivia	222.5691	220.0411	217.68	215.371	213.048	210.8598	208.9232	207.1963	205.5859	204.299
Cambodia	303.747	297.5214	292.1625	287.3685	282.9461	279.1401	276.3574	274.2152	272.3828	270.8922
Cameroon	292.3333	288.471	284.2885	279.2154	273.757	267.7385	261.7222	256.1282	250.5943	244.6605
Cape Verde	190.4052	189.6477	188.8948	188.2539	187.5644	186.1392	184.5257	182.9646	183.5097	182.2188
Congo	390.3861	383.7722	383.9513	367.7473	362.4706	355.4336	354.1594	355.7912	345.6894	344.0942
Cote d'Ivoire	351.2499	344.4579	339.4691	334.9584	331.8479	327.8073	323.2328	317.2701	310.1642	303.7401
Djibouti	283.3361	279.9791	276.4172	272.5447	269.4192	266.2792	264.9038	262.1243	260.3392	258.0371
Egypt	534.5218	543.227	544.7561	537.5572	538.4219	523.5111	518.4283	526.4097	524.8218	525.4322
El Salvador	172.7639	176.0688	170.6302	170.2625	161.1912	163.542	171.2515	171.6802	169.1992	167.2945
Federated States of Micronesia	466.1099	465.9515	465.1991	463.0164	460.794	459.3947	458.9149	458.0429	456.2349	454.3434
Georgia	495.7689	512.6867	509.1728	496.0685	477.1479	465.1826	465.9318	478.0931	494.5972	496.218
Ghana	328.6522	326.6943	324.2312	321.8694	317.6749	313.4601	309.4467	306.1699	302.6388	298.2453
Honduras	249.6281	247.5155	246.1876	246.4754	245.7406	245.7757	246.7132	244.6593	242.4481	240.2083
India	273.8964	276.1286	279.7247	283.1571	284.8791	284.0366	283.6081	283.7759	285.4779	282.2795
Indonesia	350.231	351.5678	351.7459	351.0077	351.044	350.7424	350.6392	350.4106	346.5785	342.8637
Kenya	249.2997	246.6038	243.4232	240.1317	236.1149	231.4124	227.3674	224.3147	221.6553	218.6366
Kiribati	455.9842	456.474	456.437	454.6247	452.3546	449.4588	446.4586	442.8095	438.6427	434.6567
Kyrgyzstan	544.1123	525.0605	511.4205	507.0409	502.5452	493.9719	488.2011	468.974	444.5354	436.3624
Laos	453.7888	442.5779	430.4047	416.8301	407.2574	398.2473	389.5245	381.2277	373.9311	368.1114
Lesotho	453.5195	449.2284	449.9865	444.8947	445.6901	446.5795	442.5871	437.0814	421.7798	405.1258
Mauritania	255.7619	252.2385	248.273	246.0107	243.9253	241.7317	238.6505	236.0511	234.2017	232.3472
Moldova	510.7564	511.2774	508.191	469.7604	452.9716	433.2756	441.0284	444.7515	421.5451	408.5022
Mongolia	596.4328	602.0047	576.9589	550.8118	519.5054	495.7508	477.532	468.4741	463.4737	460.0425
Morocco	478.7461	470.4318	463.6244	457.0317	446.8833	440.9674	435.3462	429.6343	424.3626	419.1459
Myanmar	256.4158	247.712	238.53	231.3134	224.5763	219.204	213.7463	209.6985	205.7248	202.1045
Nicaragua	164.5267	158.9291	150.5357	150.2216	149.4522	149.0553	152.6146	144.7268	139.9402	137.0156
Niger	240.8886	239.2353	237.0105	236.902	236.3888	236.8384	238.0233	238.9589	239.709	238.3388
Pakistan	441.6631	438.3956	435.6016	433.4756	431.7924	430.4417	429.7905	427.9218	426.1258	423.0313
Papua New Guinea	608.2924	604.9783	601.3912	596.7077	591.0478	585.278	578.7851	572.5156	566.8814	561.4939

Philippines	366.848	372.2705	372.4654	374.1948	376.9526	377.7464	376.4228	380.6781	375.7538	370.4369
Sao Tome and Principe	266.3571	267.1728	267.8744	268.0859	271.7353	270.2204	268.668	268.2818	270.4426	270.1134
Solomon Islands	490.4858	488.0578	484.965	480.1324	475.7745	471.9291	468.5747	465.4847	462.2649	459.7797
Sri Lanka	255.5667	239.8355	235.9763	232.5272	225.3993	216.2508	212.1026	207.0822	201.5169	197.0932
Sudan	487.2046	479.9594	472.7865	464.592	457.8668	451.4415	445.5574	440.1916	435.255	431.388
Timor-Leste	329.1698	326.5054	324.5805	324.0812	324.3211	327.1619	331.2601	332.8059	333.634	335.3455
Tunisia	345.612	339.9647	334.6828	331.4693	329.1376	327.268	325.5178	323.5379	321.4525	318.9908
Ukraine	637.6527	587.0583	571.125	546.2865	542.8165	537.4405	510.3686	583.2711	565.452	539.8491
Uzbekistan	791.3349	781.987	778.8175	771.3129	772.2716	770.6447	754.771	746.7353	734.7666	724.4167
Vanuatu	566.9193	563.1561	558.5023	555.3158	553.0342	550.7217	549.0249	548.4805	547.4542	546.3002
Vietnam	270.151	266.3665	262.4189	258.2632	254.7256	251.968	249.9834	248.1991	246.6714	245.4646
Zambia	266.9862	261.5001	259.8848	255.6341	250.8982	246.4702	242.4111	239.3056	236.3192	234.4993

Age Standardized Death rates- Low Income countries (2008-2017)

Country name	2008	2009	2010	2011	2012	2013	2014	2015	2016	2017
Afghanistan	693.4487	677.8455	662.6169	649.7256	637.1616	626.1621	616.6694	608.3015	601.5117	597.0293
Benin	252.7368	251.6936	250.5106	250.7337	250.7267	248.419	245.3664	242.0932	238.9727	235.8481
Burkina Faso	280.005	282.0726	280.2483	279.2169	279.6888	278.8668	277.4883	275.2915	272.4182	269.0485
Burundi	328.6147	321.0395	317.004	313.4533	309.8407	305.667	301.6341	298.479	294.9723	293.0677
Central African Republic	450.7134	449.8535	449.9551	448.0681	444.1785	445.3755	444.5944	443.6055	440.7928	435.7265
Chad	292.5967	293.8994	295.2447	294.4838	292.8466	289.2615	287.2985	285.0532	283.4847	280.9955
Comoros	281.5765	277.8206	274.3455	272.5655	270.2107	268.2238	266.5606	264.7805	263.0312	261.5161
Democratic Republic of the Congo	343.14	341.7417	340.0489	339.2778	337.8007	333.6378	329.4388	325.4805	321.8132	318.9486
Eritrea	352.536	348.3402	346.4384	340.9149	334.8275	329.1927	324.1291	323.7582	317.4691	311.1095
Ethiopia	236.157	226.5897	216.2545	208.8918	202.2597	195.0445	190.8937	187.6971	184.7659	182.6344
Guinea	334.5195	338.3559	342.0297	343.282	344.6042	345.3296	346.6671	344.5728	341.075	336.7168
Guinea-Bissau	429.1474	425.9462	420.2665	413.3254	408.997	404.2759	399.49	394.6129	388.9557	382.4743
Haiti	459.9272	455.4801	454.9508	453.8324	449.4536	444.915	440.822	437.1738	433.9231	430.5481
Liberia	283.2795	285.287	286.0436	284.4704	284.538	282.6085	279.1656	276.8746	274.7672	272.5093
Madagascar	433.4851	433.1648	431.6945	429.0223	425.4296	421.5882	417.8032	414.3495	410.0928	405.9941
Malawi	263.111	256.6373	252.8621	247.3722	242.7028	238.6362	235.1066	232.5338	230.2834	227.349
Mali	280.3852	278.2097	276.9527	274.6051	273.0865	272.1028	271.2565	270.4916	270.1558	268.0242
Mozambique	362.6849	361.8582	362.0908	359.7808	356.8653	352.2	347.5739	342.5625	336.7356	329.9419
Nepal	251.7777	254.8869	257.4135	261.5119	263.7801	264.131	263.0943	263.5303	262.5492	260.7973
Niger	240.8886	239.2353	237.0105	236.902	236.3888	236.8384	238.0233	238.9589	239.709	238.3388
North Korea	337.4986	334.9067	333.312	331.2953	329.9587	329.3702	327.7425	326.8726	324.5389	321.6811
Rwanda	218.6219	210.3697	205.2244	201.0809	198.4842	197.1778	195.6722	193.9295	192.5048	191.3751
Senegal	244.6496	242.9339	243.1681	243.9498	243.7776	243.7486	244.2638	244.4243	243.3064	241.2191
Sierra Leone	359.2842	358.7388	358.5212	354.766	351.1285	343.9521	338.3256	334.2617	329.8887	325.721
South Sudan	305.0772	300.2835	296.0392	292.2696	289.5652	287.3772	284.802	283.84	282.5393	280.7752
Syria	393.9888	387.1346	381.907	379.1242	376.9906	378.0718	378.8141	377.6273	377.9392	376.2643
Tajikistan	434.3167	431.578	430.0232	429.703	424.9035	420.4208	420.6292	426.4648	428.9046	427.6982
Tanzania	225.4218	223.1444	221.1739	219.0279	219.7144	220.385	219.7232	219.1048	218.1718	217.2882
The Gambia	341.1653	340.5569	339.6928	339.6445	339.5753	339.1222	337.9238	336.2641	333.819	331.4303
Togo	310.3391	307.7683	306.3768	303.6182	300.5483	296.3888	291.6926	287.0873	283.3324	280.0328
Uganda	259.3489	250.064	241.3351	234.2956	229.8062	227.3158	224.3182	219.4865	215.4124	213.3327
Yemen	520.2505	512.9382	504.6396	499.3245	495.0052	491.6074	490.1485	491.3076	492.9732	495.0027

Zimbabwe	399.0601	397.3711	387.696	370.4478	354.9099	341.3103	330.5272	320.9852	313.6081	307.8462
