# Supplementary material for: Disparities in Cardiovascular Research Output and Disease Outcomes among High-, Middle- and Low-Income Countries – An Analysis of Global Cardiovascular Publications over the Last Decade (2008–2017)
Source: Glob Heart. 2021 Jan 18;16(1):4. doi: 10.5334/gh.815 (PMC7845477; doi:10.5334/gh.815)
Supplement: Appendix F. — Total Population for countries in each income group (2008–2017). [file gh-16-1-815-s6.pdf]

Total population- High income countries (2008-2017)

Country name	2008	2009	2010	2011	2012	2013	2014	2015	2016	2017
Andorra	83861	84462	84449	83751	82431	80788	79223	78014	77281	76965
Antigua and Barbuda	92478	93581	94661	95719	96777	97824	98875	99923	100963	102012
Argentina	40382389	40799407	41223889	41656879	42096739	42539925	42981515	43417765	43847430	44271041
Australia	21249200	21691700	22031750	22340024	22742475	23145901	23504138	23850784	24210809	24598933
Austria	8321496	8343323	8363404	8391643	8429991	8479823	8546356	8642699	8736668	8809212
Bahrain	1114590	1185029	1240862	1278269	1300217	1315411	1336397	1371855	1425171	1492584
Barbados	277319	278470	279569	280601	281585	282509	283385	284217	284996	285719
Belgium	10709973	10796493	10895586	11047744	11128246	11182817	11209057	11274196	11331422	11372068
Brunei	379252	383772	388662	394013	399748	405716	411704	417542	423196	428697
Canada	33245773	33628571	34005274	34342780	34750545	35152370	35535348	35832513	36264604	36708083
Chile	16661942	16829442	16993354	17153357	17309746	17462982	17613798	17762681	17909754	18054726
Croatia	4434508	4429078	4417781	4280622	4267558	4255689	4238389	4203604	4174349	4125700
Cyprus	1081563	1098076	1112607	1124835	1135062	1143896	1152309	1160985	1170125	1179551
Czech Republic	10384603	10443936	10474410	10496088	10510785	10514272	10525347	10546059	10566332	10591323
Denmark	5493621	5523095	5547683	5570572	5591572	5614932	5643475	5683483	5728010	5769603
Estonia	1337090	1334515	1331475	1327439	1322696	1317997	1314545	1315407	1315790	1315480
Finland	5313399	5338871	5363352	5388272	5413971	5438972	5461512	5479531	5495303	5511303
Germany	82110097	81902307	81776930	80274983	80425823	80645605	80982500	81686611	82348669	82695000
Greece	11077841	11107017	11121341	11104899	11045011	10965211	10892413	10820883	10775971	10760421
Hungary	10038188	10022650	10000023	9971727	9920362	9893082	9866468	9843028	9814023	9781127
Iceland	317414	318499	318041	319014	320716	323764	327386	330815	335439	341284
Ireland	4489544	4535375	4560155	4580084	4599533	4623816	4657740	4701957	4755335	4813608
Israel	7308800	7485600	7623600	7765800	7910500	8059500	8215700	8380100	8546000	8712400
Italy	58826731	59095365	59277417	59379449	59539717	60233948	60789140	60730582	60627498	60551416
Japan	1.28E+08	1.28E+08	1.28E+08	1.28E+08	1.28E+08	1.27E+08	1.27E+08	1.27E+08	1.27E+08	1.27E+08
Kuwait	2652340	2818939	2998083	3191051	3395556	3598385	3782450	3935794	4052584	4136528
Latvia	2177322	2141669	2097555	2059709	2034319	2012647	1993782	1977527	1959537	1940740
Lithuania	3198231	3162916	3097282	3028115	2987773	2957689	2932367	2904910	2868231	2827721
Luxembourg	488650	497783	506953	518347	530946	543360	556319	569604	582014	599449
Malta	409379	412477	414508	416268	420028	425967	434558	445053	455356	465292
Netherlands	16445593	16530388	16615394	16693074	16754962	16804432	16865008	16939923	17030314	17132854
New Zealand	4259800	4302600	4350700	4384000	4408100	4442100	4509700	4595700	4693200	4793900

Norway	4768212	4828726	4889252	4953088	5018573	5079623	5137232	5190239	5234519	5282223
Oman	2759014	2882942	3041460	3237268	3464644	3711481	3960925	4199810	4424762	4636262
Panama	3516268	3579385	3643222	3707782	3772938	3838462	3903986	3969249	4034119	4098587
Poland	38125759	38151603	38042794	38063255	38063164	38040196	38011735	37986412	37970087	37975841
Portugal	10558177	10568247	10573100	10557560	10514844	10457295	10401062	10358076	10325452	10293718
Qatar	1389342	1590780	1779676	1952054	2109568	2250473	2374419	2481539	2569804	2639211
Saudi Arabia	25940770	26661492	27425676	28238020	29086357	29944476	30776722	31557144	32275687	32938213
Seychelles	86956	87298	89770	87441	88303	89949	91359	93419	94677	95843
Singapore	4839396	4987573	5076732	5183688	5312437	5399162	5469724	5535002	5607283	5612253
Slovakia	5379233	5386406	5391428	5398384	5407579	5413393	5418649	5423801	5430798	5439892
Slovenia	2021316	2039669	2048583	2052843	2057159	2059953	2061980	2063531	2065042	2066748
Spain	45954106	46362946	46576897	46742697	46773055	46620045	46480882	46444832	46484062	46572028
Sweden	9219637	9298515	9378126	9449213	9519374	9600379	9696110	9799186	9923085	10067744
Switzerland	7647675	7743831	7824909	7912398	7996861	8089346	8188649	8282396	8373338	8466017
The Bahamas	348676	354856	360832	366568	372039	377240	382169	386838	391232	395361
Trinidad and Tobago	1315372	1321618	1328100	1334788	1341588	1348248	1354493	1360092	1364962	1369125
United Arab Emirates	6894278	7666393	8270684	8672475	8900453	9006263	9070867	9154302	9269612	9400145
United Kingdom	61806995	62276270	62766365	63258918	63700300	64128226	64613160	65128861	65595565	66022273
United States	3.04E+08	3.07E+08	3.09E+08	3.12E+08	3.14E+08	3.16E+08	3.19E+08	3.21E+08	3.23E+08	3.26E+08
Uruguay	3350824	3362755	3374415	3385624	3396777	3408005	3419546	3431552	3444006	3456750

Total population- Upper Middle income countries (2008-2017)

country name	2008	2009	2010	2011	2012	2013	2014	2015	2016	2017
Albania	2947314	2927519	2913021	2905195	2900401	2895092	2889104	2880703	2876101	2873457
Algeria	34860715	35465760	36117637	36819558	37565847	38338562	39113313	39871528	40606052	41318142
Armenia	2908220	2888584	2877311	2875581	2881922	2893509	2906220	2916950	2924816	2930450
Azerbaijan	8763400	8947243	9054332	9173082	9295784	9416801	9535079	9649341	9757812	9862429
Belarus	9527985	9506765	9490583	9473172	9464495	9465997	9474511	9489616	9501534	9507875
Belize	306165	313929	321608	329192	336701	344181	351694	359288	366954	374681
Bosnia and Herzegovina	3763599	3746561	3722084	3688865	3648200	3604999	3566002	3535961	3516816	3507017
Botswana	1946351	1979882	2014866	2051339	2089315	2128507	2168573	2209197	2250260	2291661
Brazil	1.93E+08	1.95E+08	1.97E+08	1.99E+08	2.01E+08	2.02E+08	2.04E+08	2.06E+08	2.08E+08	2.09E+08
Bulgaria	7492561	7444443	7395599	7348328	7305888	7265115	7223938	7177991	7127822	7075991
China	1.32E+09	1.33E+09	1.34E+09	1.34E+09	1.35E+09	1.36E+09	1.36E+09	1.37E+09	1.38E+09	1.39E+09
Colombia	44901544	45416181	45918097	46406646	46881475	47342981	47791911	48228697	48653419	49065615
Costa Rica	4429508	4488263	4545280	4600474	4654122	4706401	4757575	4807852	4857274	4905769
Cuba	11309754	11318602	11333051	11354651	11382146	11412167	11439767	11461432	11475982	11484636
Dominica	71074	71229	71440	71718	72044	72400	72778	73162	73543	73925
Dominican Republic	9636520	9767758	9897985	10027095	10154950	10281296	10405844	10528394	10648791	10766998
Ecuador	14447562	14691275	14934690	15177355	15419666	15661547	15903112	16144368	16385068	16624858
Equatorial Guinea	868418	909111	951104	994290	1038593	1083746	1129424	1175389	1221490	1267689
Fiji	843340	851967	859950	867086	873596	879715	885806	892149	898760	905502
Gabon	1536411	1586754	1640210	1697101	1756817	1817271	1875713	1930175	1979786	2025137
Grenada	103930	104296	104677	105075	105481	105909	106360	106823	107317	107825
Guatemala	14006366	14316208	14630417	14948919	15271056	15596214	15923559	16252429	16582469	16913503
Guyana	746314	745693	746556	749100	753091	758081	763393	768514	773303	777859
Iran	72845542	73687565	74567511	75491582	76453574	77435384	78411092	79360487	80277428	81162788
Iraq	29111417	29894652	30762701	31727053	32776571	33883145	35006080	36115649	37202572	38274618
Jamaica	2790122	2804082	2817210	2829493	2840992	2851807	2862087	2871934	2881355	2890299
Jordan	6489822	6821116	7182390	7574943	7992573	8413464	8809306	9159302	9455802	9702353
Kazakhstan	15674000	16092822	16321872	16557201	16792089	17035550	17288285	17542806	17794055	18037646
Libya	6053078	6121053	6169140	6193501	6198258	6195970	6204108	6234955	6293253	6374616
Macedonia	2067378	2069093	2070739	2072383	2074036	2075739	2077495	2079308	2081206	2083160
Malaysia	27111069	27605383	28112289	28635128	29170456	29706724	30228017	30723155	31187265	31624264
Maldives	345054	354501	364511	375131	386203	397397	408247	418403	427756	436330

Marshall Islands	52218	52320	52425	52542	52663	52793	52898	52994	53066	53127
Mauritius	1244121	1247429	1250400	1252404	1255882	1258653	1260934	1262605	1263473	1264613
Mexico	1.14E+08	1.16E+08	1.17E+08	1.19E+08	1.21E+08	1.23E+08	1.24E+08	1.26E+08	1.28E+08	1.29E+08
Montenegro	616969	618294	619428	620079	620601	621207	621810	622159	622303	622471
Namibia	2106375	2137040	2173170	2215621	2263934	2316520	2370992	2425561	2479713	2533794
Paraguay	6047117	6127837	6209877	6293783	6379219	6465740	6552584	6639119	6725308	6811297
Peru	28641980	29001507	29373646	29759989	30158966	30565716	30973354	31376671	31773839	32165485
Romania	20537875	20367487	20246871	20147528	20058035	19983693	19908979	19815481	19702332	19586539
Russian Federation	1.43E+08	1.43E+08	1.43E+08	1.43E+08	1.43E+08	1.44E+08	1.44E+08	1.44E+08	1.44E+08	1.44E+08
Saint Lucia	169220	171022	172580	173832	174835	175660	176421	177206	178015	178844
Saint Vincent and the Grenadines	109165	109253	109315	109341	109328	109320	109357	109455	109643	109897
Samoa	183526	184826	186205	187665	189194	190757	192290	193759	195125	196440
Serbia	7350222	7320807	7291436	7234099	7199077	7164132	7130576	7095383	7058322	7022268
South Africa	50412129	50970818	51584663	52263516	52998213	53767396	54539571	55291225	56015473	56717156
Suriname	515148	520619	526103	531589	537077	542540	547928	553208	558368	563402
Thailand	66545760	66881867	67208808	67530130	67843979	68143065	68416772	68657600	68863514	69037513
Tonga	103005	103604	104137	104577	104951	105328	105782	106364	107122	108020
Turkey	70440032	71339185	72326914	73409455	74569867	75787333	77030628	78271472	79512426	80745020
Turkmenistan	4935762	5007950	5087210	5174061	5267839	5366277	5466241	5565284	5662544	5758075
Venezuela	28141701	28587323	29028033	29463291	29893080	30317848	30738378	31155134	31568179	31977065

Total population- Lower Middle income countries (2008-2017)

country name	2008	2009	2010	2011	2012	2013	2014	2015	2016	2017
Angola	21759420	22549547	23369131	24218565	25096150	25998340	26920466	27859305	28813463	29784193
Bangladesh	1.49E+08	1.5E+08	1.52E+08	1.54E+08	1.56E+08	1.58E+08	1.59E+08	1.61E+08	1.63E+08	1.65E+08
Bhutan	700950	714458	727641	740510	752967	764961	776448	787386	797765	807610
Bolivia	9599855	9758748	9918242	10078343	10239004	10400264	10562159	10724705	10887882	11051600
Cambodia	13880509	14090208	14308740	14537886	14776866	15022692	15270790	15517635	15762370	16005373
Cameroon	18907008	19432541	19970495	20520447	21082383	21655715	22239904	22834522	23439189	24053727
Cape Verde	491723	496963	502384	508067	513979	520106	526437	532913	539560	546388
Congo	4115435	4253712	4386693	4512730	4633363	4751393	4871101	4995648	5125821	5260750
Cote d'Ivoire	19497986	19936366	20401331	20895311	21418603	21966312	22531350	23108472	23695919	24294750
Djibouti	822934	836840	851146	865937	881185	896688	912164	927414	942333	956985
Egypt	80953881	82465022	84107606	85897561	87813257	89807433	91812566	93778172	95688681	97553151
El Salvador	6110301	6137276	6164626	6192560	6221246	6250777	6281189	6312478	6344722	6377853
Federated States of Micronesia	104478	103960	103616	103468	103503	103702	104015	104433	104937	105544
Georgia	4030000	3978000	3926000	3875000	3825000	3776000	3727000	3717100	3719300	3717100
Ghana	23298640	23903831	24512104	25121796	25733049	26346251	26962563	27582821	28206728	28833629
Honduras	7872658	8035021	8194778	8351600	8505646	8657785	8809216	8960829	9112867	9265067
India	1.2E+09	1.21E+09	1.23E+09	1.25E+09	1.26E+09	1.28E+09	1.29E+09	1.31E+09	1.32E+09	1.34E+09
Indonesia	2.36E+08	2.39E+08	2.43E+08	2.46E+08	2.49E+08	2.52E+08	2.55E+08	2.58E+08	2.61E+08	2.64E+08
Kenya	39148416	40237204	41350152	42486839	43646629	44826849	46024250	47236259	48461567	49699862
Kiribati	98440	100568	102652	104656	106613	108535	110458	112407	114395	116398
Kyrgyzstan	5318700	5383300	5447900	5514600	5607200	5719600	5835500	5956900	6079500	6201500
Laos	6052190	6152036	6246274	6333487	6415169	6494557	6576397	6663967	6758353	6858160
Lesotho	1999930	2019209	2040551	2064166	2089928	2117361	2145785	2174645	2203821	2233339
Mauritania	3407541	3506288	3609543	3717672	3830239	3946170	4063920	4182341	4301018	4420184
Moldova	3570108	3565604	3562045	3559986	3559519	3558566	3556397	3554108	3551954	3549750
Mongolia	2628131	2668289	2712650	2761516	2814226	2869107	2923896	2976877	3027398	3075647
Morocco	31596855	31989897	32409639	32858823	33333789	33824769	34318082	34803322	35276786	35739580
Myanmar	49479752	49800690	50155896	50553031	50986514	51448196	51924182	52403669	52885223	53370609
Nicaragua	5594506	5666581	5737723	5807820	5877108	5945747	6013997	6082035	6149928	6217581
Nigeria	1.5E+08	1.54E+08	1.59E+08	1.63E+08	1.67E+08	1.72E+08	1.76E+08	1.81E+08	1.86E+08	1.91E+08
Pakistan	1.64E+08	1.67E+08	1.71E+08	1.74E+08	1.78E+08	1.82E+08	1.86E+08	1.89E+08	1.93E+08	1.97E+08
Papua New Guinea	6787187	6947447	7108239	7269348	7430836	7592865	7755785	7919825	8084991	8251162

Philippines	90751864	92220879	93726624	95277940	96866642	98481032	1E+08	1.02E+08	1.03E+08	1.05E+08
Sao Tome and Principe	166913	170813	174776	178800	182889	187045	191266	195553	199910	204327
Solomon Islands	504477	516079	527790	539614	551531	563513	575504	587482	599419	611343
Sri Lanka	19945832	20075086	20198353	20315017	20425000	20585000	20771000	20966000	21203000	21444000
Sudan	32955496	33650619	34385963	35167314	35990192	36849918	37737913	38647803	39578828	40533330
Timor-Leste	1078110	1092021	1109591	1131523	1156760	1184366	1212814	1240977	1268671	1296311
Tunisia	10407336	10521834	10639931	10761467	10886668	11014558	11143908	11273661	11403248	11532127
Ukraine	46258200	46053300	45870700	45706100	45593300	45489600	45271947	45154029	45004645	44831159
Uzbekistan	27302800	27767400	28562400	29339400	29774500	30243200	30757700	31298900	31847900	32387200
Vanuatu	225340	230785	236295	241871	247485	253142	258850	264603	270402	276244
Vietnam	86707801	87565407	88472512	89436644	90451881	91497725	92544915	93571567	94569072	95540800
Zambia	13082517	13456417	13850033	14264756	14699937	15153210	15620974	16100587	16591390	17094130

Total population- Low income countries (2008-2017)

country name	2008	2009	2010	2011	2012	2013	2014	2015	2016	2017
Afghanistan	27294031	28004331	28803167	29708599	30696958	31731688	32758020	33736494	34656032	35530081
Benin	8696916	8944706	9199259	9460802	9729160	10004451	10286712	10575952	10872298	11175692
Burkina Faso	14689726	15141099	15605217	16081904	16571216	17072723	17585977	18110624	18646433	19193382
Burundi	8212264	8489031	8766930	9043508	9319710	9600186	9891790	10199270	10524117	10864245
Central African Republic	4345386	4404230	4448525	4476153	4490416	4499653	4515392	4546100	4594621	4659080
Chad	11133861	11502786	11887202	12288651	12705135	13133589	13569438	14009413	14452543	14899994
Comoros	657229	673252	689692	706569	723868	741500	759385	777424	795601	813912
Democratic republic of Congo	60373608	62409435	64523263	66713597	68978682	71316033	73722860	76196619	78736153	81339988
Eritrea	4232636	4310334	4390840	4474690	4474690	4474690	4474690	4474690	4474690	4474690
Ethiopia	83184892	85416253	87702670	90046756	92444183	94887724	97366774	99873033	1.02E+08	1.05E+08
Guinea	10323142	10556524	10794170	11035170	11281469	11536615	11805509	12091533	12395924	12717176
Guinea-Bissau	1480841	1517448	1555880	1596154	1638139	1681495	1725744	1770526	1815698	1861283
Haiti	9705029	9852870	9999617	10145054	10289210	10431776	10572466	10711061	10847334	10981229
Liberia	3662993	3811528	3948125	4070167	4181563	4286291	4390737	4499621	4613823	4731906
Madagascar	19996469	20569121	21151640	21743949	22346573	22961146	23589801	24234088	24894551	25570895
Malawi	14271234	14714602	15167095	15627618	16097305	16577147	17068838	17573607	18091575	18622104
Mali	14138216	14606597	15075085	15540989	16006670	16477818	16962846	17467905	17994837	18541980
Mozambique	22846758	23524063	24221405	24939005	25676606	26434372	27212382	28010691	28829476	29668834
Nepal	26475859	26741103	27023137	27327147	27649925	27985310	28323241	28656282	28982771	29304998
Niger	15228525	15813913	16425578	17064636	17731634	18426372	19148219	19896965	20672987	21477348
North Korea	24335146	24463021	24591599	24722298	24854034	24985976	25116363	25243917	25368620	25490965
Rwanda	9708169	9977446	10246842	10516071	10788853	11065151	11345357	11629553	11917508	12208407
Senegal	12203957	12550917	12916229	13300910	13703513	14120320	14546111	14976994	15411614	15850567
Sierra Leone	6165372	6310260	6458720	6611692	6766103	6922079	7079162	7237025	7396190	7557212
South Sudan	9263136	9670667	10067192	10448857	10818258	11177490	11530971	11882136	12230730	12575714
Syria	20325443	20824893	21018834	20863993	20420701	19809141	19203090	18734987	18430453	18269868
Tajikistan	7309728	7472819	7641630	7815949	7995062	8177809	8362745	8548651	8734951	8921343
Tanzania	43270144	44664231	46098591	47570902	49082997	50636595	52234869	53879957	55572201	57310019
The Gambia	1588572	1639560	1692149	1746363	1802125	1859324	1917852	1977590	2038501	2100568
Togo	6161796	6330472	6502952	6679282	6859482	7042948	7228915	7416802	7606374	7797694
Uganda	31663896	32771895	33915133	35093648	36306796	37553726	38833338	40144870	41487965	42862958
Yemen	22356391	22974929	23606779	24252206	24909969	25576322	26246327	26916207	27584213	28250420

Zimbabwe	13558469	13810599	14086317	14386649	14710826	15054506	15411675	15777451	16150362	16529904
