# Supplementary material for: Disparities in Cardiovascular Research Output and Disease Outcomes among High-, Middle- and Low-Income Countries – An Analysis of Global Cardiovascular Publications over the Last Decade (2008–2017)
Source: Glob Heart. 2021 Jan 18;16(1):4. doi: 10.5334/gh.815 (PMC7845477; doi:10.5334/gh.815)
Supplement: Appendix G. — Human Development Index for countries in each income group (2008–2017). [file gh-16-1-815-s7.pdf]

Human Development Index- High Income countries (2008-2017)

Country	2008	2009	2010	2011	2012	2013	2014	2015	2016	2017
Andorra	0.831	0.83	0.828	0.827	0.849	0.85	0.853	0.854	0.856	0.858
Antigua and Barbuda	0.777	0.77	0.766	0.762	0.765	0.768	0.77	0.775	0.778	0.78
Argentina	0.795	0.799	0.813	0.819	0.818	0.82	0.82	0.822	0.822	0.825
Australia	0.92	0.921	0.923	0.925	0.929	0.931	0.933	0.936	0.938	0.939
Austria	0.884	0.886	0.895	0.897	0.899	0.897	0.901	0.903	0.906	0.908
Bahrain	0.796	0.794	0.796	0.798	0.8	0.807	0.81	0.832	0.846	0.846
Barbados	0.781	0.783	0.782	0.787	0.795	0.796	0.796	0.797	0.799	0.8
Belgium	0.899	0.899	0.903	0.904	0.905	0.908	0.909	0.913	0.915	0.916
Brunei	0.84	0.842	0.842	0.846	0.852	0.853	0.853	0.852	0.852	0.853
Canada	0.899	0.899	0.902	0.905	0.908	0.911	0.918	0.92	0.922	0.926
Chile	0.806	0.804	0.808	0.814	0.819	0.828	0.833	0.84	0.842	0.843
Croatia	0.803	0.802	0.808	0.815	0.816	0.821	0.824	0.827	0.828	0.831
Cyprus	0.852	0.856	0.85	0.853	0.852	0.853	0.856	0.86	0.867	0.869
Czech Republic	0.854	0.857	0.862	0.865	0.865	0.874	0.879	0.882	0.885	0.888
Denmark	0.909	0.906	0.91	0.922	0.924	0.931	0.928	0.926	0.928	0.929
Estonia	0.841	0.839	0.845	0.853	0.859	0.862	0.864	0.866	0.868	0.871
Finland	0.904	0.899	0.903	0.907	0.908	0.912	0.914	0.915	0.918	0.92
Germany	0.917	0.917	0.921	0.926	0.928	0.928	0.93	0.933	0.934	0.936
Greece	0.857	0.858	0.856	0.852	0.854	0.856	0.864	0.866	0.868	0.87
Hungary	0.818	0.818	0.823	0.827	0.83	0.835	0.833	0.834	0.835	0.838
Iceland	0.89	0.89	0.891	0.901	0.909	0.92	0.925	0.927	0.933	0.935
Ireland	0.908	0.906	0.909	0.895	0.902	0.911	0.921	0.929	0.934	0.938
Israel	0.88	0.882	0.887	0.892	0.893	0.895	0.899	0.901	0.902	0.903
Italy	0.868	0.868	0.87	0.875	0.874	0.876	0.874	0.876	0.878	0.88
Japan	0.881	0.88	0.885	0.89	0.895	0.899	0.903	0.905	0.907	0.909
Kuwait	0.789	0.79	0.792	0.794	0.796	0.795	0.799	0.802	0.804	0.803
Latvia	0.821	0.818	0.816	0.821	0.824	0.833	0.838	0.841	0.844	0.847
Lithuania	0.831	0.822	0.824	0.828	0.831	0.836	0.851	0.852	0.855	0.858
Luxembourg	0.89	0.883	0.889	0.892	0.892	0.892	0.895	0.899	0.904	0.904
Malta	0.829	0.83	0.843	0.843	0.849	0.856	0.862	0.871	0.875	0.878
Netherlands	0.906	0.906	0.91	0.921	0.921	0.923	0.924	0.926	0.928	0.931
New Zealand	0.894	0.898	0.899	0.902	0.905	0.907	0.91	0.914	0.915	0.917

Norway	0.938	0.938	0.942	0.943	0.942	0.946	0.946	0.948	0.951	0.953
Oman	0.782	0.789	0.793	0.795	0.804	0.812	0.815	0.822	0.822	0.821
Panama	0.755	0.756	0.758	0.764	0.771	0.776	0.781	0.781	0.785	0.789
Poland	0.824	0.828	0.835	0.839	0.836	0.85	0.842	0.855	0.86	0.865
Portugal	0.814	0.817	0.822	0.826	0.829	0.837	0.839	0.842	0.845	0.847
Qatar	0.835	0.833	0.825	0.836	0.844	0.854	0.853	0.854	0.855	0.856
Saudi Arabia	0.791	0.796	0.808	0.823	0.835	0.844	0.852	0.854	0.854	0.853
Seychelles	0.741	0.741	0.747	0.741	0.77	0.779	0.786	0.791	0.793	0.797
Singapore	0.883	0.884	0.909	0.914	0.92	0.923	0.928	0.929	0.93	0.932
Slovakia	0.822	0.824	0.829	0.837	0.842	0.844	0.845	0.851	0.853	0.855
Slovenia	0.878	0.878	0.882	0.884	0.877	0.885	0.887	0.889	0.894	0.896
Spain	0.856	0.858	0.865	0.87	0.873	0.875	0.88	0.885	0.889	0.891
Sweden	0.901	0.899	0.905	0.906	0.908	0.912	0.92	0.929	0.932	0.933
Switzerland	0.917	0.927	0.932	0.932	0.935	0.938	0.939	0.942	0.943	0.944
The Bahamas	0.79	0.787	0.789	0.793	0.807	0.807	0.807	0.807	0.806	0.807
Trinidad and Tobago	0.773	0.772	0.775	0.773	0.774	0.779	0.779	0.783	0.785	0.784
United Arab Emirates	0.838	0.835	0.836	0.841	0.846	0.851	0.855	0.86	0.862	0.863
United Kingdom	0.896	0.899	0.905	0.899	0.898	0.915	0.919	0.918	0.92	0.922
United States	0.91	0.91	0.914	0.917	0.918	0.916	0.918	0.92	0.922	0.924
Uruguay	0.766	0.778	0.773	0.782	0.79	0.797	0.801	0.8	0.802	0.804

Human Development Index- Upper Middle Income countries (2008-2017)

Country name	2008	2009	2010	2011	2012	2013	2014	2015	2016	2017
Albania	0.724	0.729	0.741	0.752	0.767	0.771	0.773	0.776	0.782	0.785
Algeria	0.709	0.719	0.729	0.736	0.74	0.745	0.747	0.749	0.753	0.754
Armenia	0.727	0.724	0.728	0.731	0.737	0.742	0.745	0.748	0.749	0.755
Azerbaijan	0.719	0.736	0.74	0.741	0.745	0.752	0.758	0.758	0.757	0.757
Belarus	0.774	0.784	0.792	0.798	0.803	0.804	0.807	0.805	0.805	0.808
Belize	0.7	0.699	0.699	0.702	0.706	0.705	0.706	0.709	0.709	0.708
Bosnia and Herzegovina	0.719	0.72	0.713	0.721	0.739	0.747	0.754	0.755	0.766	0.768
Botswana	0.639	0.648	0.66	0.673	0.683	0.693	0.701	0.706	0.712	0.717
Brazil	0.716	0.718	0.727	0.731	0.736	0.748	0.752	0.757	0.758	0.759
Bulgaria	0.771	0.774	0.779	0.782	0.786	0.792	0.797	0.807	0.81	0.813
China	0.685	0.694	0.706	0.714	0.722	0.729	0.738	0.743	0.748	0.752
Colombia	0.71	0.715	0.719	0.725	0.725	0.735	0.738	0.742	0.747	0.747
Costa Rica	0.75	0.752	0.754	0.76	0.772	0.776	0.78	0.788	0.791	0.794
Cuba	0.78	0.782	0.779	0.778	0.767	0.765	0.768	0.772	0.774	0.777
Dominica	0.72	0.72	0.722	0.722	0.721	0.721	0.724	0.721	0.718	0.715
Dominican Republic	0.695	0.696	0.703	0.706	0.71	0.713	0.718	0.729	0.733	0.736
Ecuador	0.711	0.712	0.715	0.721	0.726	0.734	0.742	0.743	0.749	0.752
Equatorial Guinea	0.586	0.589	0.581	0.584	0.589	0.59	0.59	0.593	0.593	0.591
Fiji	0.703	0.707	0.711	0.717	0.719	0.727	0.73	0.738	0.738	0.741
Gabon	0.653	0.661	0.665	0.67	0.678	0.687	0.693	0.694	0.698	0.702
Grenada	0.739	0.738	0.743	0.747	0.749	0.754	0.761	0.767	0.77	0.772
Guatemala	0.598	0.604	0.611	0.619	0.613	0.616	0.643	0.645	0.649	0.65
Guyana	0.621	0.624	0.63	0.639	0.642	0.645	0.648	0.651	0.652	0.654
Iran	0.741	0.747	0.755	0.766	0.781	0.784	0.788	0.789	0.796	0.798
Iraq	0.643	0.646	0.649	0.656	0.659	0.666	0.666	0.668	0.672	0.685
Jamaica	0.708	0.709	0.712	0.715	0.721	0.726	0.728	0.73	0.732	0.732
Jordan	0.736	0.734	0.728	0.726	0.726	0.727	0.73	0.733	0.735	0.735
Kazakhstan	0.759	0.763	0.765	0.772	0.781	0.788	0.793	0.797	0.797	0.8
Libya	0.757	0.755	0.755	0.707	0.741	0.707	0.695	0.694	0.693	0.706
Macedonia	0.728	0.731	0.735	0.738	0.74	0.743	0.747	0.754	0.756	0.757
Malaysia	0.761	0.765	0.772	0.778	0.781	0.785	0.79	0.795	0.799	0.802
Maldives	0.66	0.66	0.671	0.682	0.688	0.696	0.705	0.71	0.712	0.717

Marshall Islands	.	.	.	0.708	0.708	0.708	0.708	0.708	0.708	0.708
Mauritius	0.734	0.741	0.749	0.758	0.767	0.772	0.782	0.782	0.788	0.79
Mexico	0.742	0.743	0.743	0.751	0.757	0.756	0.761	0.767	0.772	0.774
Montenegro	0.786	0.788	0.793	0.798	0.8	0.803	0.805	0.809	0.81	0.814
Namibia	0.575	0.583	0.594	0.607	0.617	0.628	0.636	0.642	0.645	0.647
Paraguay	0.664	0.659	0.675	0.68	0.68	0.695	0.698	0.702	0.702	0.702
Peru	0.712	0.715	0.717	0.729	0.729	0.736	0.746	0.745	0.748	0.75
Romania	0.795	0.798	0.797	0.798	0.795	0.8	0.802	0.805	0.807	0.811
Russian Federation	0.774	0.771	0.78	0.789	0.798	0.804	0.807	0.813	0.815	0.816
Saint Lucia	0.719	0.724	0.731	0.734	0.73	0.733	0.737	0.744	0.745	0.747
Saint Vincent and the Grenadines	0.71	0.713	0.715	0.717	0.718	0.721	0.72	0.72	0.721	0.723
Samoa	0.685	0.687	0.693	0.697	0.697	0.7	0.703	0.706	0.711	0.713
Serbia	0.757	0.759	0.759	0.769	0.768	0.771	0.775	0.78	0.785	0.787
South Africa	0.633	0.642	0.649	0.657	0.664	0.675	0.685	0.692	0.696	0.699
Suriname	0.691	0.677	0.703	0.706	0.711	0.715	0.718	0.722	0.719	0.72
Thailand	0.714	0.718	0.724	0.727	0.731	0.728	0.735	0.741	0.748	0.755
Tonga	0.702	0.706	0.712	0.716	0.717	0.716	0.717	0.721	0.724	0.726
Turkey	0.71	0.718	0.734	0.753	0.76	0.771	0.778	0.783	0.787	0.791
Turkmenistan			0.673	0.68	0.686	0.692	0.697	0.701	0.705	0.706
Venezuela	0.753	0.753	0.759	0.771	0.774	0.776	0.778	0.775	0.766	0.761

Human Development Index- Lower Middle Income countries (2008-2017)

Country name	2008	2009	2010	2011	2012	2013	2014	2015	2016	2017
Angola	0.502	0.522	0.52	0.535	0.543	0.554	0.564	0.572	0.577	0.581
Bangladesh	0.523	0.535	0.545	0.557	0.567	0.575	0.583	0.592	0.597	0.608
Bhutan	0.543	0.554	0.566	0.575	0.585	0.589	0.599	0.603	0.609	0.612
Bolivia	0.64	0.647	0.649	0.655	0.662	0.668	0.675	0.681	0.689	0.693
Cambodia	0.521	0.521	0.537	0.546	0.553	0.56	0.566	0.571	0.576	0.582
Cameroon	0.492	0.499	0.506	0.515	0.526	0.535	0.543	0.548	0.553	0.556
Cape Verde	0.625	0.626	0.629	0.635	0.636	0.642	0.644	0.647	0.652	0.654
Congo	0.536	0.55	0.557	0.56	0.573	0.582	0.595	0.613	0.612	0.606
Cote d'Ivoire	0.43	0.437	0.442	0.445	0.454	0.462	0.465	0.478	0.486	0.492
Djibouti	0.435	0.441	0.449	0.454	0.459	0.463	0.467	0.47	0.474	0.476
Egypt	0.658	0.66	0.665	0.668	0.675	0.68	0.683	0.691	0.694	0.696
El Salvador	0.659	0.659	0.671	0.666	0.67	0.671	0.67	0.674	0.679	0.674
Federated States of Micronesia	0.596	0.603	0.608	0.613	0.616	0.619	0.618	0.627	0.627	0.627
Georgia	0.728	0.731	0.735	0.741	0.75	0.757	0.765	0.771	0.776	0.78
Ghana	0.542	0.547	0.554	0.563	0.57	0.577	0.576	0.585	0.588	0.592
Honduras	0.59	0.591	0.596	0.598	0.597	0.6	0.603	0.609	0.614	0.617
India	0.564	0.57	0.581	0.591	0.6	0.607	0.618	0.627	0.636	0.64
Indonesia	0.646	0.656	0.661	0.669	0.675	0.681	0.683	0.686	0.691	0.694
Kenya	0.523	0.533	0.543	0.552	0.559	0.566	0.572	0.578	0.585	0.59
Kiribati	0.585	0.584	0.59	0.59	0.598	0.609	0.616	0.621	0.61	0.612
Kyrgyzstan	0.631	0.636	0.636	0.639	0.649	0.658	0.663	0.666	0.669	0.672
Laos	0.529	0.539	0.546	0.558	0.569	0.579	0.586	0.593	0.598	0.601
Lesotho	0.48	0.489	0.493	0.498	0.505	0.505	0.509	0.511	0.516	0.52
Mauritania	0.476	0.484	0.487	0.49	0.499	0.508	0.514	0.514	0.516	0.52
Moldova	0.666	0.662	0.67	0.677	0.684	0.693	0.696	0.693	0.697	0.7
Mongolia	0.683	0.689	0.697	0.711	0.72	0.729	0.734	0.737	0.743	0.741
Morocco	0.602	0.608	0.616	0.626	0.635	0.645	0.65	0.655	0.662	0.667
Myanmar	0.509	0.519	0.53	0.54	0.549	0.558	0.564	0.569	0.574	0.578
Nicaragua	0.614	0.615	0.621	0.627	0.633	0.639	0.649	0.652	0.657	0.658
Nigeria	0.485	0.49	0.484	0.494	0.512	0.519	0.524	0.527	0.53	0.532
Pakistan	0.515	0.522	0.526	0.53	0.535	0.538	0.548	0.551	0.56	0.562
Papua New Guinea	0.503	0.51	0.52	0.529	0.53	0.534	0.536	0.542	0.543	0.544

Philippines	0.661	0.659	0.665	0.67	0.677	0.685	0.689	0.693	0.696	0.699
Sao Tome and Principe	0.53	0.538	0.542	0.548	0.551	0.56	0.567	0.58	0.584	0.589
Solomon Islands	0.504	0.497	0.507	0.514	0.529	0.539	0.539	0.546	0.543	0.546
Sri Lanka	0.738	0.74	0.745	0.751	0.757	0.759	0.763	0.766	0.768	0.77
Sudan	0.463	0.468	0.47	0.474	0.485	0.475	0.492	0.497	0.499	0.502
Timor-Leste	0.599	0.61	0.619	0.624	0.599	0.614	0.61	0.63	0.631	0.625
Tunisia	0.707	0.71	0.716	0.718	0.719	0.723	0.725	0.728	0.732	0.735
Ukraine	0.733	0.727	0.733	0.738	0.743	0.745	0.748	0.743	0.746	0.751
Uzbekistan	0.653	0.659	0.666	0.674	0.683	0.69	0.695	0.698	0.703	0.71
Vanuatu	0.589	0.59	0.591	0.592	0.592	0.597	0.598	0.599	0.6	0.603
Vietnam	0.64	0.656	0.654	0.664	0.67	0.675	0.678	0.684	0.689	0.694
Zambia	0.517	0.533	0.544	0.556	0.569	0.574	0.58	0.583	0.586	0.588

Human Development Index- Low Income countries (2008-2017)

Country name	2008	2009	2010	2011	2012	2013	2014	2015	2016	2017
Afghanistan	0.437	0.453	0.463	0.471	0.482	0.487	0.491	0.493	0.494	0.498
Benin	0.462	0.468	0.473	0.479	0.489	0.5	0.505	0.508	0.512	0.515
Burkina Faso	0.357	0.366	0.375	0.385	0.394	0.401	0.405	0.412	0.42	0.423
Burundi	0.372	0.387	0.395	0.403	0.408	0.414	0.421	0.418	0.418	0.417
Central African Republic	0.337	0.343	0.351	0.358	0.365	0.344	0.349	0.357	0.362	0.367
Chad	0.348	0.363	0.371	0.382	0.391	0.397	0.403	0.407	0.405	0.404
Comoros	0.47	0.476	0.482	0.487	0.493	0.499	0.501	0.502	0.502	0.503
Democratic Republic of Congo	0.39	0.397	0.407	0.415	0.42	0.426	0.436	0.444	0.452	0.457
Eritrea	0.407	0.416	0.416	0.417	0.422	0.425	0.428	0.433	0.436	0.44
Ethiopia	0.394	0.401	0.412	0.423	0.43	0.438	0.445	0.451	0.457	0.463
Guinea	0.396	0.399	0.404	0.418	0.428	0.435	0.44	0.443	0.449	0.459
Guinea-Bissau	0.415	0.421	0.426	0.435	0.437	0.44	0.445	0.449	0.453	0.455
Haiti	0.466	0.47	0.47	0.477	0.481	0.486	0.49	0.493	0.496	0.498
Liberia	0.399	0.404	0.407	0.417	0.42	0.429	0.431	0.432	0.432	0.435
Madagascar	0.5	0.503	0.504	0.504	0.507	0.509	0.512	0.514	0.517	0.519
Malawi	0.417	0.431	0.441	0.45	0.455	0.461	0.468	0.47	0.474	0.477
Mali	0.39	0.398	0.403	0.408	0.408	0.408	0.414	0.418	0.421	0.427
Mozambique	0.388	0.397	0.403	0.407	0.412	0.423	0.427	0.432	0.435	0.437
Nepal	0.502	0.514	0.529	0.535	0.548	0.554	0.56	0.566	0.569	0.574
Niger	0.303	0.308	0.318	0.325	0.336	0.34	0.345	0.347	0.351	0.354
North Korea	0.874	0.869	0.884	0.888	0.89	0.893	0.896	0.898	0.9	0.903
Rwanda	0.455	0.47	0.485	0.493	0.5	0.503	0.509	0.51	0.52	0.524
Senegal	0.445	0.449	0.456	0.467	0.476	0.481	0.486	0.492	0.499	0.505
Sierra Leone	0.373	0.381	0.385	0.392	0.407	0.419	0.423	0.413	0.413	0.419
South Sudan	.	.	0.413	0.416	0.388	0.392	0.397	0.399	0.394	0.388
Syria	0.646	0.648	0.644	0.642	0.631	0.572	0.55	0.538	0.536	0.536
Tajikistan	0.618	0.62	0.634	0.637	0.642	0.646	0.645	0.645	0.647	0.65
Tanzania	0.477	0.486	0.493	0.499	0.506	0.507	0.515	0.528	0.533	0.538
The Gambia	0.435	0.438	0.441	0.44	0.445	0.453	0.454	0.457	0.457	0.46
Togo	0.44	0.449	0.456	0.463	0.466	0.472	0.481	0.495	0.5	0.503
Uganda	0.47	0.477	0.486	0.49	0.492	0.496	0.5	0.505	0.508	0.516
Yemen	0.485	0.491	0.498	0.499	0.505	0.507	0.505	0.483	0.462	0.452

Zimbabwe	0.439	0.45	0.467	0.478	0.505	0.516	0.525	0.529	0.532	0.535
