# Supplementary material for: Disparities in Cardiovascular Research Output and Disease Outcomes among High-, Middle- and Low-Income Countries – An Analysis of Global Cardiovascular Publications over the Last Decade (2008–2017)
Source: Glob Heart. 2021 Jan 18;16(1):4. doi: 10.5334/gh.815 (PMC7845477; doi:10.5334/gh.815)
Supplement: Appendix H. — Cardiovascular publications and prevalence rates: Trends (A) and relationship (B). [file gh-16-1-815-s8.pdf]

Figure A: Trends in integer counts of cardiovascular publications and age-standardized prevalence rates of cardiovascular disease in World Bank income groups (2008-2017).

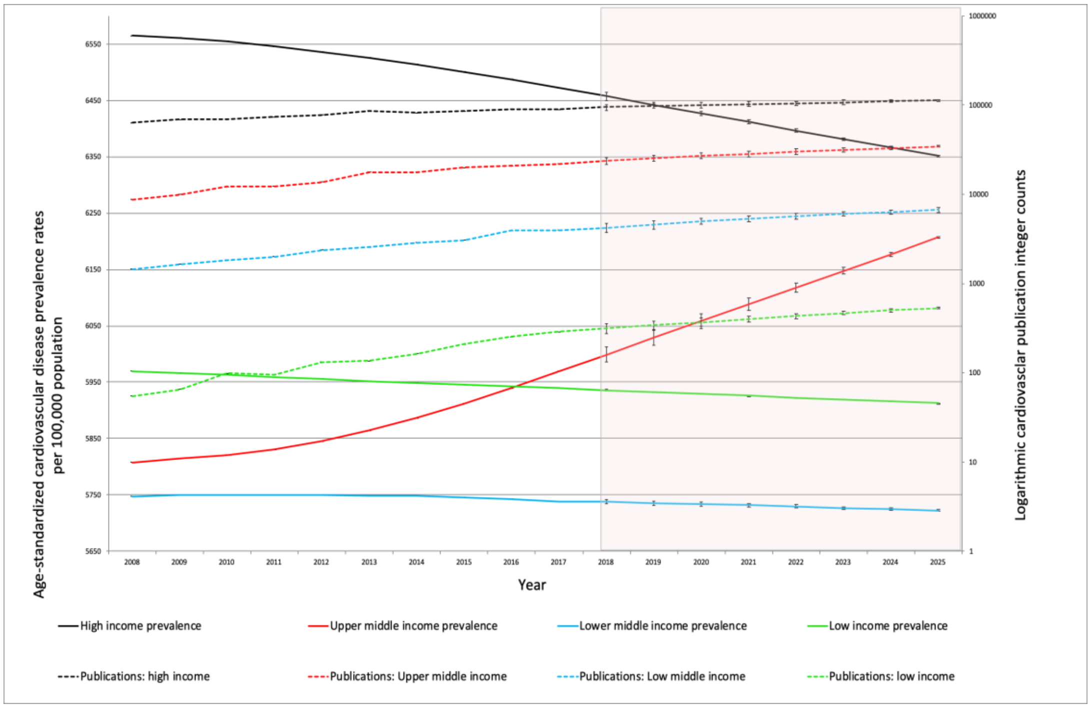

Figure B: Logarithmic cardiovascular publication integer counts against age-standardized cardiovascular disease prevalence rates per 100,000 population of for the year 2013.

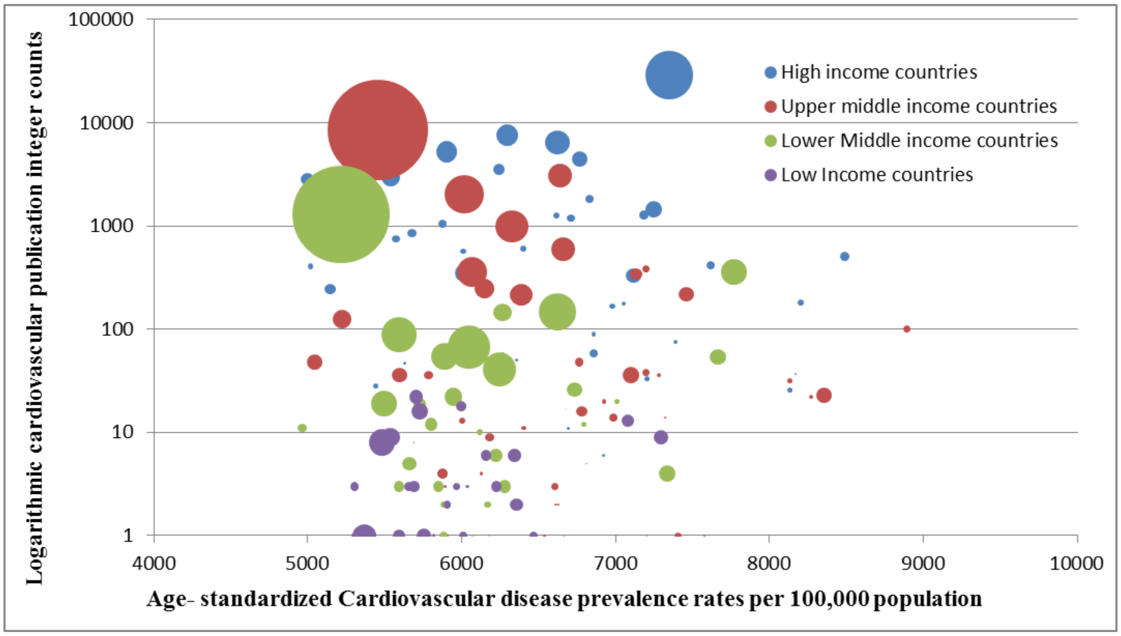

Each bubble represents a country (n=181). Size of the bubble represents the country population and color represents World Bank income group.
